# Supplementary material for: Precision gestational diabetes treatment: a systematic review and meta-analyses
Source: Commun Med (Lond). 2023 Oct 5;3:135. doi: 10.1038/s43856-023-00371-0 (PMC10550921; doi:10.1038/s43856-023-00371-0)

Supplementary Information

Precision gestational diabetes treatment: a systematic review and meta-analyses

Jamie L Benham, Véronique Gingras, Niamh-Maire McLennan, Jasper Most, Jennifer M Yamamoto, Catherine E Aiken, Susan E Ozanne, Rebecca M Reynolds, ADA/EASD PMDI

Supplementary Note 1 Search Strategy

PubMed Search

Systematic review 1: Lifestyle interventions

#1 Diabetes, Gestational"[Mesh]

#2 gestational diabetes"[Title/Abstract] OR GDM[Title/Abstract] OR pregnancy induced diabetes[Title/Abstract] OR pregnancy-induced diabetes

#3 #1 OR #2

a.#4 ((((("Body-Weight Trajectory"[Mesh]) OR "Body Mass Index"[Mesh]) OR "Body Weight"[Mesh]) OR "Body Composition"[Mesh]) OR "Waist Circumference"[Mesh]) OR weight gain[MeSH]

#5 bodyweight*[Title/Abstract] OR "body weight*"[Title/Abstract] OR body-weight*[Title/Abstract] OR bmi[Title/Abstract] OR "body mass index"[Title/Abstract] OR "body composition"[Title/Abstract] OR bodycomposition[Title/Abstract] OR body-composition[Title/Abstract] OR "body fat"[Title/Abstract] OR bodyfat body-fat[Title/Abstract] OR "waist circumfer*"[Title/Abstract] OR waistcircumfer*[Title/Abstract] OR waist-circumfer*[Title/Abstract] OR "weight gain"[Title/Abstract] OR weight-gain[Title/Abstract] OR weightgain[Title/Abstract]

#6 #4 OR #5

b. #7 ((("Diet"[Mesh]) OR "Dietary Supplements"[Mesh]) OR "Diet, Carbohydrate-Restricted"[Mesh]) OR "Diet, Fat-Restricted"[Mesh]) OR "Caloric Restriction"[Mesh] OR nutrition[MeSH]

#8 diet*[Title/Abstract] OR "caloric restrict*"[Title/Abstract] OR calory-restrict*[Title/Abstract] OR eating[Title/Abstract] OR macronutri*[Title/Abstract] OR nutrition*[Title/Abstract] OR protein*[Title/Abstract] OR meal[Title/Abstract] OR beverage*[Title/Abstract] OR meat*[Title/Abstract] OR behavior*[Title/Abstract] OR behaviour*[Title/Abstract] OR habit*[Title/Abstract] OR sleep*[Title/Abstract] OR food*[Title/Abstract]

#9 #7 OR #8

c. #10 exercise[MeSH] OR physical fitness [MeSH] OR lifestyle [MeSH] OR healthy lifestyle [MeSH] OR sedentary behavior [MeSH]

#11 exercis*[Title/Abstract] OR "physical activit*"[Title/Abstract] OR fitness[Title/Abstract] OR sedentary[Title/Abstract] OR walk*[Title/Abstract] OR stretch*[Title/Abstract] OR lifestyle*[Title/Abstract] OR "life style*"[Title/Abstract] OR life-style*[Title/Abstract] OR wellness[Title/Abstract] OR "strength train*"[Title/Abstract] OR strength-train*[Title/Abstract]

#12 #10 OR #11

#13 #6 OR #9 OR #12

#14 #3 AND #13

#15 ("controlled trial*" OR randomi* OR "observational stud*" OR RCT OR "retrospective stud*" OR Metformin in Gestational Diabetes trial) OR ("controlled trial*"[Publication Type] OR randomi*[Publication Type] OR "observational stud*"[Publication Type] OR RCT[Publication Type] OR "retrospective stud*"[Publication Type])

#16 #14 AND #15

#17 #16 filters: humans, English

Systematic review 2: Pharmacological interventions

#1 “Diabetes, Gestational"[Mesh]

#2 "gestational diabetes"[Title/Abstract] OR GDM[Title/Abstract] OR pregnancy induced diabetes[Title/Abstract] OR pregnancy-induced diabetes

#3 #1 OR #2

#4 "Insulin"[Mesh]

#5 ((("Metformin"[Mesh]) OR "Sulfonylurea Compounds"[Mesh]) OR "Glyburide"[Mesh]) OR "Secretagogues"[Mesh]

#6 insulin*[Title/Abstract] OR novolin[Title/Abstract] OR iletin[Title/Abstract] OR sulfonylurea*[Title/Abstract] OR Acetohexamide[Title/Abstract] OR Carbutamide[Title/Abstract] OR Chlorpropamide[Title/Abstract] OR Gliclazide[Title/Abstract] OR Glyburide[Title/Abstract] OR Tolazamide[Title/Abstract] OR Tolbutamide[Title/Abstract] OR sulphonylurea*[Title/Abstract] OR glibenclamide[Title/Abstract] OR secretagogues[Title/Abstract] OR “pharmacological therapy”[Title/Abstract]

#7 #4 OR #5 OR #6

#8 #3 AND #7

#9 ("controlled trial*" OR randomi* OR "observational stud*" OR RCT OR "retrospective stud*" OR Metformin in Gestational Diabetes trial) OR ("controlled trial*"[Publication Type] OR randomi*[Publication Type] OR "observational stud*"[Publication Type] OR RCT[Publication Type] OR "retrospective stud*"[Publication Type])

#10 #8 AND #9

#11 #10 Filters: humans, English

Embase search

#1 'pregnancy diabetes mellitus'/exp

#2 'gestational diabetes':ab,ti OR gdm:ab,ti OR 'pregnancy-induced diabetes':ab,ti OR 'pregnancy induced diabetes':ab,ti

#3 #1 OR #2

Systematic review 1: Lifestyle interventions

#4 'weight trajectory' AND 'body weight'/exp OR 'body mass'/exp OR 'body weight'/exp OR 'body composition'/exp OR 'waist circumference'/exp OR 'body weight gain'/exp

#5 bodyweight*:ab,ti OR 'body weight*':ab,ti OR 'body-weight*or bmi':ab,ti OR 'body mass index':ab,ti OR bodycomposition:ab,ti OR 'body composition':ab,ti OR 'body fator body-fat':ab,ti OR 'waist circumfer*':ab,ti OR 'waistcircumfer*or waist-circumfer*':ab,ti OR 'weight gain':ab,ti OR weightgain:ab,ti

#6 #4 OR #5

#7 'diet'/exp OR 'dietary supplement'/exp OR 'low carbohydrate diet'/exp OR 'low fat diet'/exp OR 'caloric restriction'/exp OR 'nutrition'/exp

#8 diet*:ab,ti OR 'caloric restrict*':ab,ti OR 'calory restrict*':ab,ti OR eating:ab,ti OR macronutri*:ab,ti OR nutrition*:ab,ti OR protein*:ab,ti OR meal:ab,ti OR beverage*:ab,ti OR meat*:ab,ti OR behavior*:ab,ti OR behaviour*:ab,ti OR habit*:ab,ti OR sleep*:ab,ti OR food*:ab,ti

#9 #7 OR #8

#10 'exercise'/exp OR 'fitness'/exp OR 'lifestyle'/exp OR 'healthy lifestyle'/exp OR 'sedentary lifestyle'/exp

#11 exercis*:ab,ti OR 'physical activit*':ab,ti OR fitness:ab,ti OR sedentary:ab,ti OR walk*:ab,ti OR stretch*:ab,ti OR lifestyle*:ab,ti OR 'life style*':ab,ti OR wellness:ab,ti OR 'strength train*':ab,ti

#12 #10 OR #11

#13 #6 OR #9 OR #12

Study design filter

#14 ((randomized:ab,ti OR randomised:ab,ti OR randomly:ab,ti OR rct:ab,ti OR 'retrospective stud*':ab,ti OR controlled) AND clinical AND trial:ab,ti OR controlled) AND trial:ab,ti OR 'randomized controlled trial'/de OR 'controlled clinical trial'/de

Combination search RQ1 Lifestyle interventions

GDM + Lifestyle interventions + study design filter

#15 #3 AND #13 AND #14

AND [embase]/lim NOT ([embase]/lim AND [medline]/lim)

Filters Human, English

NOT 'conference abstract':it

Systematic review 2: Pharmacological interventions

#16 'insulin'/exp

#17 'metformin'/exp OR 'sulfonylurea derivative'/exp OR 'glibenclamide'/exp OR 'secretagogue'/exp

#18 (insulin* OR novolin OR iletin OR sulfonylurea* OR acetohexamide OR carbutamide OR chlorpropamide OR gliclazide OR glyburide OR tolazamide OR tolbutamide OR sulphonylurea* OR glibenclamide OR secretagogues OR pharmacological) AND therapy

#19 #16 OR #17 OR #18

Combination search RQ2 Pharmacological interventions

#6 GDM + Pharmacological interventions + study design filter

#3 AND #19 AND #13

AND [embase]/lim NOT ([embase]/lim AND [medline]/lim)

Filters Human, English

NOT 'conference abstract':it

Supplementary Figure 1 Comparison of studies of whether lifestyle was adequate or inadequate according to maternal age in years

Legend. Forest plot (a) and Funnel plot (b) for included studies comparing if lifestyle was adequate (no need for escalation to pharmacological agent(s)) or not adequate (required escalation to pharmacological agent(s)).

a)

	Lifestyle Adequate			Lifestyle Not Adequate				Mean Difference
Study or Subgroup	Mean	SD	Total	Mean	SD	Total	Weight	IV, Random, 95% CI
Ares 2017	35.56	4.22	165	36.55	3.56	36	2.8%	−0.99 [−2.32, 0.34]
Bashir 2020	31.5	5.7	358	33.3	5	443	6.2%	−1.80 [−2.55, −1.05]
Benhalima 2015	31.8	4.8	456	32.5	4.7	145	5.1%	−0.70 [−1.58, 0.18]
Ducarme 2019	30.8	5.3	128	30.8	5.5	72	2.2%	0.00 [−1.57, 1.57]
Durnwald 2011	29.2	5.7	424	29.3	5.8	36	1.4%	−0.10 [−2.07, 1.87]
Gilbert 2021	33.84	5.41	178	33.39	5.27	163	3.6%	0.45 [−0.68, 1.58]
Ikenoue 2014	35.9	4.3	91	36.1	5	50	2.0%	−0.20 [−1.84, 1.44]
Ito 2016	34.4	5.6	70	33.3	5.6	32	1.1%	1.10 [−1.24, 3.44]
Kalok 2020	32	4.4	968	32.9	4.5	96	4.7%	−0.90 [−1.84, 0.04]
Koning 2016	31.6	4.9	460	32.6	5.2	360	6.7%	−1.00 [−1.70, −0.30]
Mecacci 2021	33.9	5.33	962	34.6	5.4	1012	9.3%	−0.70 [−1.17, −0.23]
Meghelli 2020	29	5.2	58	31.6	4.6	63	1.8%	−2.60 [−4.36, −0.84]
Meshel 2016	33.52	5.75	1181	35.39	4.5	143	5.7%	−1.87 [−2.68, −1.06]
Ng 2020	30.59	5.55	1281	31.93	5.69	576	8.3%	−1.34 [−1.90, −0.78]
Nishikawa 2018	32.5	0.3	472	33.6	0.7	57	13.0%	−1.10 [−1.28, −0.92]
Ouzounian 2011	31.4	5.2	1189	31.9	5.6	262	6.3%	−0.50 [−1.24, 0.24]
Souza 2019	32	6.1	273	34.1	5.7	135	3.3%	−2.10 [−3.30, −0.90]
Suhonen 2008	33.5	5.4	520	34	5.4	385	6.6%	−0.50 [−1.21, 0.21]
Sun 2021	29.5	5	616	30.7	4.1	92	4.8%	−1.20 [−2.13, −0.27]
Wong 2011	30.9	5.4	289	31.9	5.3	323	5.3%	−1.00 [−1.85, −0.15]
Total (95% CI)			10139			4481	100.0%	−0.98 [−1.23, −0.73]

Heterogeneity: $Tau^2 = 0.12$; $Chi^2 = 35.97$, df = 19 ($P = 0.01$); $I^2 = 47\%$

Test for overall effect: Z = 7.66 ($P < 0.00001$)

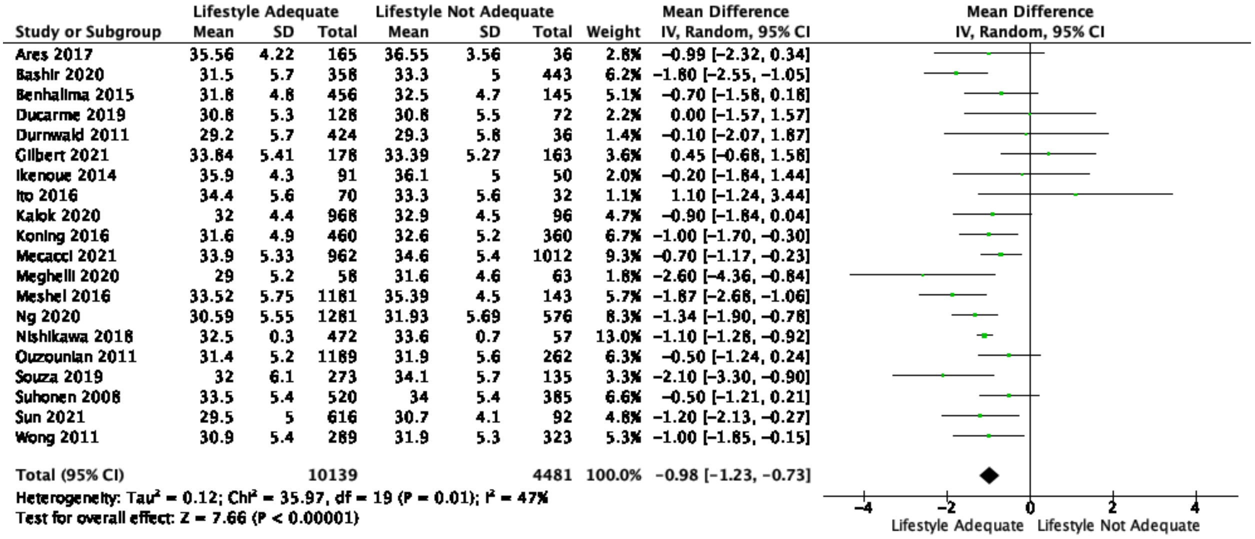

b)

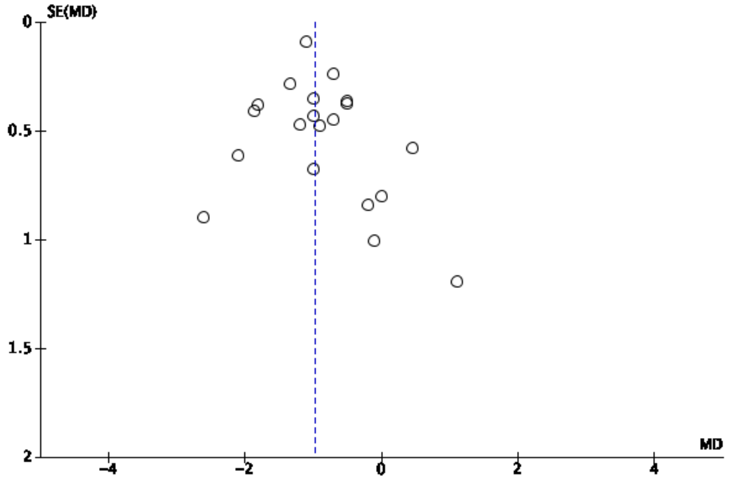

Supplementary Figure 2 Comparison of studies of whether lifestyle was adequate or inadequate according to nulliparity

Legend. Forest plot (a) and Funnel plot (b) for included studies comparing if lifestyle was adequate (no need for escalation to pharmacological agent(s)) or not adequate (required escalation to pharmacological agent(s)).

a)

	Lifestyle Adequate		Lifestyle Not Adequate			Odds Ratio
Study or Subgroup	Events	Total	Events	Total	Weight	M-H, Random, 95% CI
Benhalima 2015	163	456	34	145	10.9%	1.82 [1.18, 2.79]
Ducarme 2019	48	126	23	72	7.5%	1.31 [0.71, 2.42]
Koning 2016	223	460	110	360	14.3%	2.14 [1.60, 2.86]
Mecacci 2021	590	962	578	1012	17.0%	1.19 [0.99, 1.43]
Ouzounian 2011	497	1189	77	262	14.3%	1.73 [1.29, 2.31]
Souza 2019	74	273	24	135	9.1%	1.72 [1.03, 2.88]
Suhonen 2008	187	520	91	385	14.1%	1.81 [1.35, 2.44]
Wong 2011	85	289	101	323	12.9%	0.92 [0.65, 1.29]
Total (95% CI)		4275		2694	100.0%	1.53 [1.23, 1.89]
Total events	1867		1038			

Heterogeneity: $Tau^2 = 0.06$; $Chi^2 = 23.47$, df = 7 (P = 0.001); $I^2 = 70\%$
Test for overall effect: Z = 3.87 (P = 0.0001)

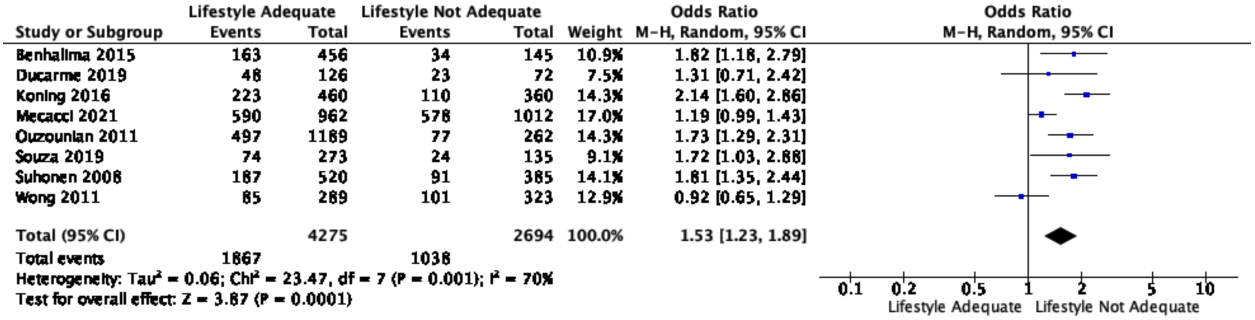

b)

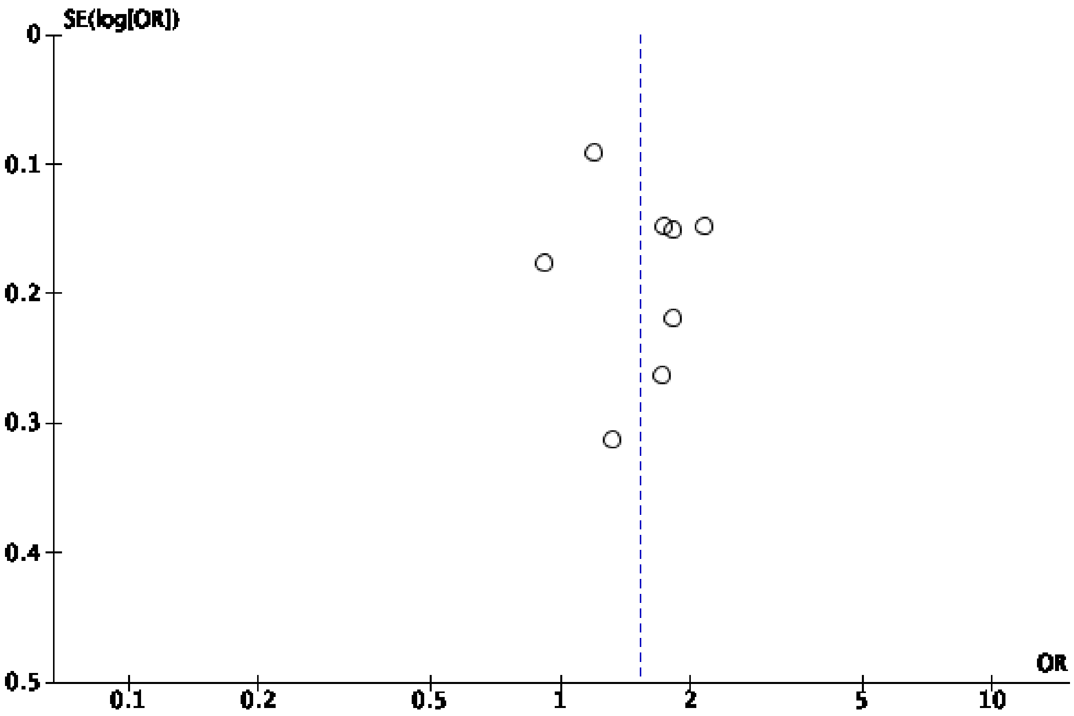

Supplementary Figure 3 Comparison of studies of whether lifestyle was adequate or inadequate according to body mass index in kg/m^2

Legend. Forest plot (a) and Funnel plot (b) for included studies comparing if lifestyle was adequate (no need for escalation to pharmacological agent(s)) or not adequate (required escalation to pharmacological agent(s)).

a)

	Lifestyle Adequate			Lifestyle Not Adequate				Mean Difference
Study or Subgroup	Mean	SD	Total	Mean	SD	Total	Weight	IV, Random, 95% CI
Ares 2017	26.82	4.94	165	27.04	6.46	36	3.1%	−0.22 [−2.46, 2.02]
Barnes 2013	24.9	5.9	1171	28.62	6.76	524	8.2%	−3.72 [−4.39, −3.05]
Bashir 2020	31.2	6	358	32.9	6.3	443	7.5%	−1.70 [−2.55, −0.85]
Benhalima 2015	26.8	12.9	456	29.1	20.2	145	1.6%	−2.30 [−5.79, 1.19]
Ducarme 2019	27.4	5.8	126	28.6	6	72	4.4%	−1.20 [−2.92, 0.52]
Durnwald 2011	29.9	4.9	424	32.7	5.8	36	3.8%	−2.80 [−4.75, −0.85]
Ikenoue 2014	20.5	3.6	91	21.1	2.6	50	6.7%	−0.60 [−1.63, 0.43]
Ito 2016	23.3	3.6	70	24.6	4.3	32	4.4%	−1.30 [−3.01, 0.41]
Mecacci 2021	23.9	4.91	962	25.2	5.49	1012	9.0%	−1.30 [−1.76, −0.84]
Meghelli 2020	43.6	2.6	58	44	2.9	63	7.0%	−0.40 [−1.38, 0.58]
Ng 2020	29	7.42	1281	31.2	7.66	576	7.9%	−2.20 [−2.95, −1.45]
Nishikawa 2018	22.41	0.21	472	23.89	0.7	57	9.6%	−1.48 [−1.66, −1.30]
Souza 2019	29.8	6.9	273	32.2	6.4	135	5.5%	−2.40 [−3.75, −1.05]
Suhonen 2008	26.1	5.4	520	29	6.8	385	7.6%	−2.90 [−3.72, −2.08]
Sun 2021	23.9	4	616	24.8	4.3	92	7.1%	−0.90 [−1.83, 0.03]
Wong 2011	26.5	6.3	289	29.9	7.3	323	6.6%	−3.40 [−4.48, −2.32]
Total (95% CI)			7332			3981	100.0%	−1.83 [−2.32, −1.35]

Heterogeneity: $Tau^2 = 0.62$; $Chi^2 = 80.60$, df = 15 ($P < 0.00001$); $I^2 = 81\%$
Test for overall effect: Z = 7.45 ($P < 0.00001$)

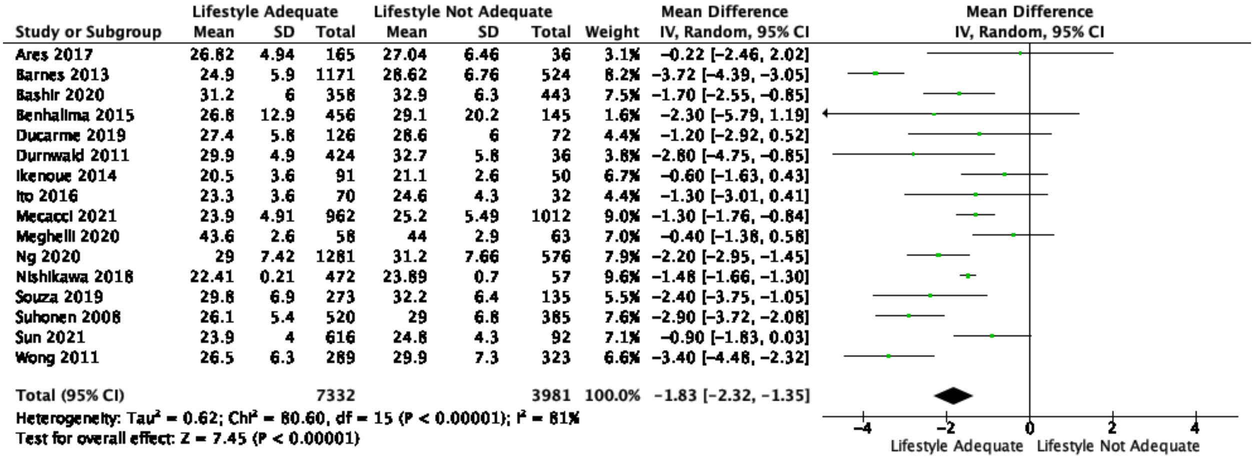

b)

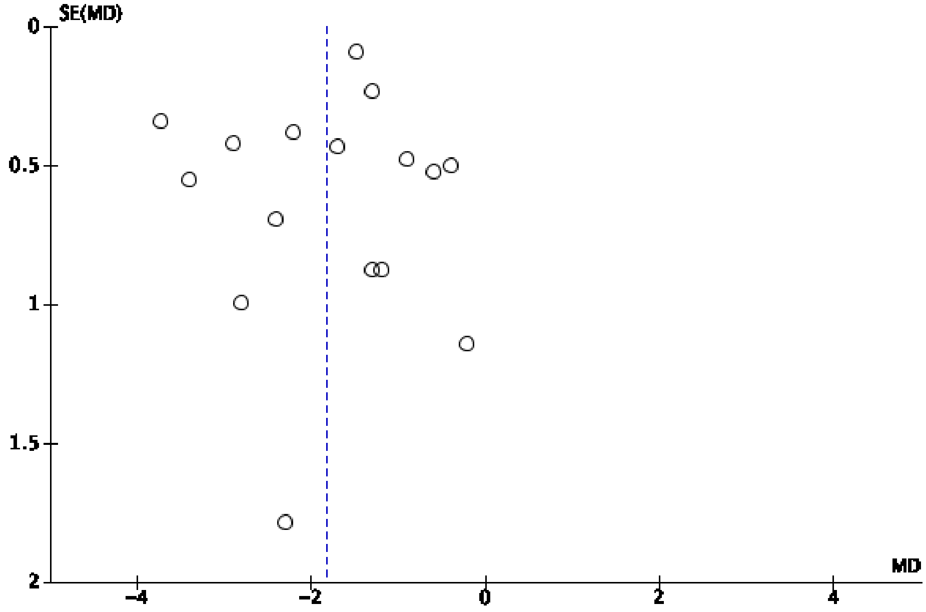

Supplementary Figure 4 Comparison of studies of whether lifestyle was adequate or inadequate according to previous history of gestational diabetes

Legend. Forest plot (a) and Funnel plot (b) for included studies comparing if lifestyle was adequate (no need for escalation to pharmacological agent(s)) or not adequate (required escalation to pharmacological agent(s)).

a)

Study or Subgroup	Lifestyle Adequate Events	Lifestyle Adequate Total	Lifestyle Not Adequate Events	Lifestyle Not Adequate Total	Weight	Odds Ratio M-H, Random, 95% CI
Benhalima 2015	47	456	31	145	8.4%	0.42 [0.26, 0.70]
Ducarme 2019	23	126	14	72	5.6%	0.93 [0.44, 1.94]
Ikenoue 2014	1	91	0	50	0.5%	1.67 [0.07, 41.86]
Kalok 2020	151	968	29	96	8.8%	0.43 [0.27, 0.68]
Koning 2016	25	460	61	360	8.6%	0.28 [0.17, 0.46]
Krispin 2021	61	314	76	328	10.2%	0.80 [0.55, 1.17]
Mecacci 2021	85	962	151	1012	11.8%	0.55 [0.42, 0.73]
Meghelli 2020	6	58	24	63	3.8%	0.19 [0.07, 0.50]
Meshel 2016	293	1181	55	143	10.5%	0.53 [0.37, 0.76]
Nishikawa 2018	25	472	5	57	3.7%	0.58 [0.21, 1.58]
Ouzounian 2011	185	1189	89	262	11.5%	0.36 [0.27, 0.48]
Souza 2019	17	273	32	135	6.7%	0.21 [0.11, 0.40]
Wong 2011	48	289	84	323	9.9%	0.57 [0.38, 0.84]
Total (95% CI)		6839		3046	100.0%	0.46 [0.37, 0.57]
Total events	967		651			

Heterogeneity: $Tau^2 = 0.09$; $Chi^2 = 31.08$, $df = 12$ ($P = 0.002$); $I^2 = 61\%$
Test for overall effect: $Z = 6.86$ ($P < 0.00001$)

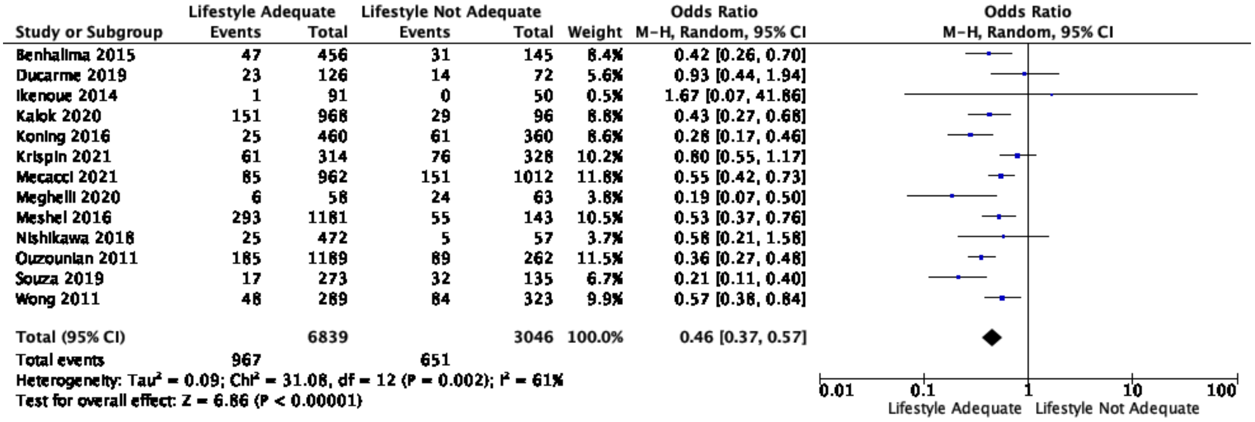

b)

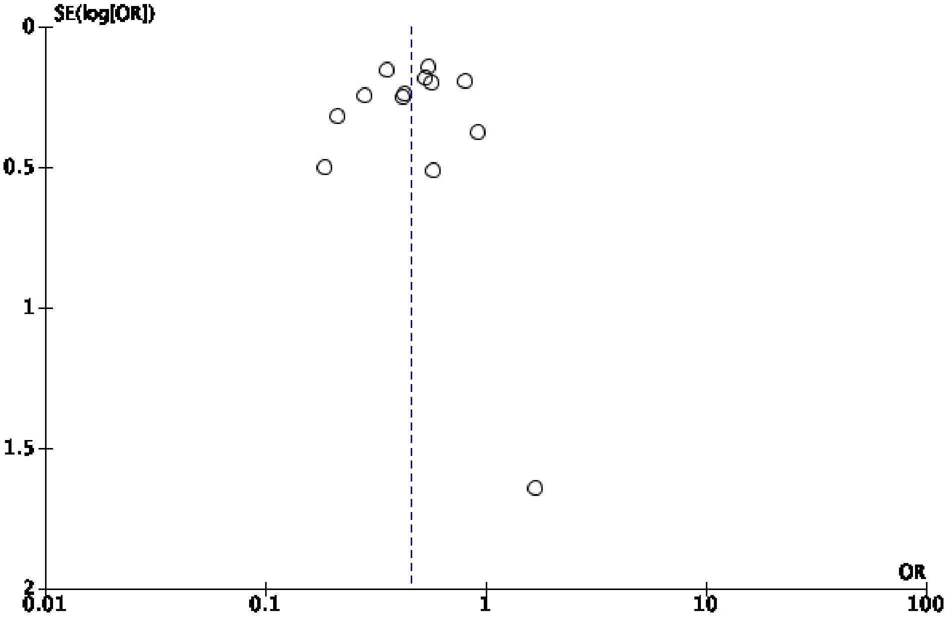

Supplementary Figure 5 Comparison of studies of whether lifestyle was adequate or inadequate according to haemoglobin A1C in %

Legend. Forest plot (a) and Funnel plot (b) for included studies comparing if lifestyle was adequate (no need for escalation to pharmacological agent(s)) or not adequate (required escalation to pharmacological agent(s)).

a)

	Lifestyle Adequate			Lifestyle Not Adequate				Mean Difference
Study or Subgroup	Mean	SD	Total	Mean	SD	Total	Weight	IV, Random, 95% CI
Barnes 2013	5.2	0.6	1171	5.5	0.6	524	14.3%	−0.30 [−0.36, −0.24]
Benhalima 2015	5.2	0.5	456	5.5	0.6	145	11.1%	−0.30 [−0.41, −0.19]
Ducarme 2019	5.2	0.3	126	5.3	0.4	72	11.3%	−0.10 [−0.21, 0.01]
Gilbert 2021	5.3	0.31	178	5.5	0.51	163	12.3%	−0.20 [−0.29, −0.11]
Ikenoue 2014	5.4	0.3	91	5.4	0.3	50	11.4%	0.00 [−0.10, 0.10]
Nishikawa 2018	5.24	0.01	472	5.46	0.06	57	16.5%	−0.22 [−0.24, −0.20]
Sun 2021	5.2	0.4	616	5.6	0.6	92	9.9%	−0.40 [−0.53, −0.27]
Wong 2011	5.41	0.39	269	5.54	0.61	323	13.1%	−0.13 [−0.21, −0.05]
Total (95% CI)			3399			1426	100.0%	−0.21 [−0.27, −0.14]

Heterogeneity: $Tau^2 = 0.01$; $Chi^2 = 43.53$, df = 7 ($P < 0.00001$); $I^2 = 84\%$
Test for overall effect: Z = 6.44 ($P < 0.00001$)

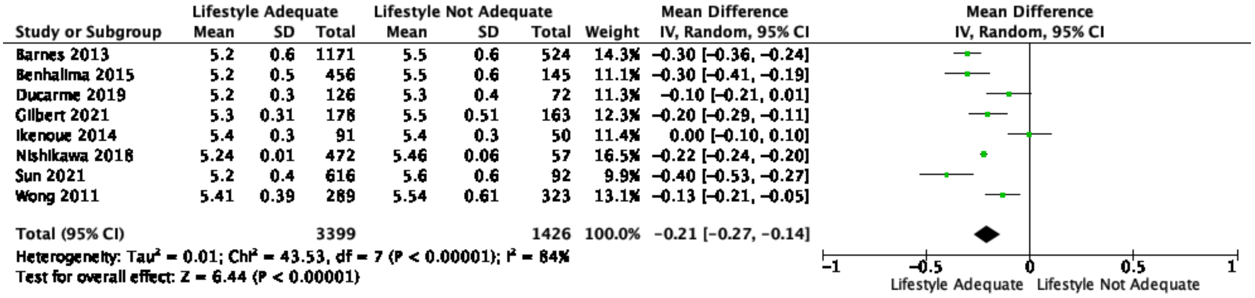

b)

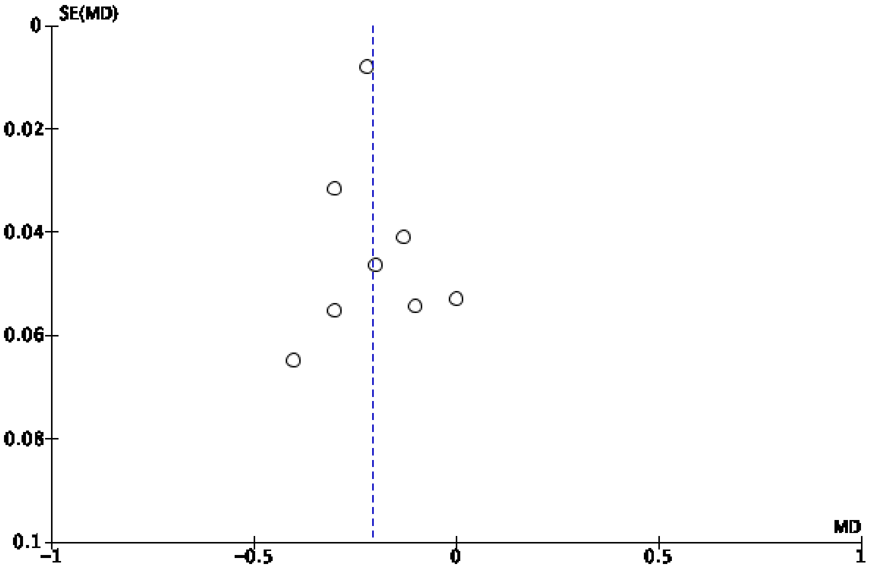

Supplementary Figure 6 Comparison of studies of whether lifestyle was adequate or inadequate according to fasting glucose in mg/dL

Legend. Forest plot (a) and Funnel plot (b) for included studies comparing if lifestyle was adequate (no need for escalation to pharmacological agent(s)) or not adequate (required escalation to pharmacological agent(s)).

a)

	Lifestyle Adequate			Lifestyle Not Adequate				Mean Difference
Study or Subgroup	Mean	SD	Total	Mean	SD	Total	Weight	IV, Random, 95% CI
Ares 2017	87.9	9.02	165	95.68	11.26	36	6.8%	−7.78 [−11.71, −3.85]
Bashir 2020	86.4	9	358	97.2	12.6	443	8.4%	−10.80 [−12.30, −9.30]
Benhalima 2015	87.7	10.3	456	97.6	18.8	145	7.3%	−9.90 [−13.10, −6.70]
Ducarme 2019	92	8	126	95	12	72	7.4%	−3.00 [−6.10, 0.10]
Ikenoue 2014	88.9	7.4	91	89.3	7.1	50	7.8%	−0.40 [−2.89, 2.09]
Ito 2016	89.6	8.7	70	91.5	7.9	32	7.2%	−1.90 [−5.31, 1.51]
Kalok 2020	84.78	10.8	968	94.5	13.32	96	7.6%	−9.72 [−12.47, −6.97]
Mecacci 2021	86.71	23.39	962	90.07	9.7	1012	8.3%	−3.36 [−4.95, −1.77]
Meshel 2016	88.09	11.91	1181	100.31	10.86	143	8.2%	−12.22 [−14.13, −10.31]
Nishikawa 2018	91.5	0.4	472	94.1	1.4	57	8.7%	−2.60 [−2.97, −2.23]
Souza 2019	96.8	5.2	273	101.1	7.8	135	8.4%	−4.30 [−5.75, −2.85]
Sun 2021	84.6	13.14	616	100.98	26.64	92	5.6%	−16.38 [−21.92, −10.84]
Wong 2011	97.38	7.02	289	99.72	10.98	323	8.4%	−2.34 [−3.79, −0.89]
Total (95% CI)			6027			2636	100.0%	−6.26 [−8.44, −4.08]

Heterogeneity: $Tau^2 = 14.18$; $Chi^2 = 265.84$, df = 12 ($P < 0.00001$); $I^2 = 95\%$
Test for overall effect: Z = 5.63 ($P < 0.00001$)

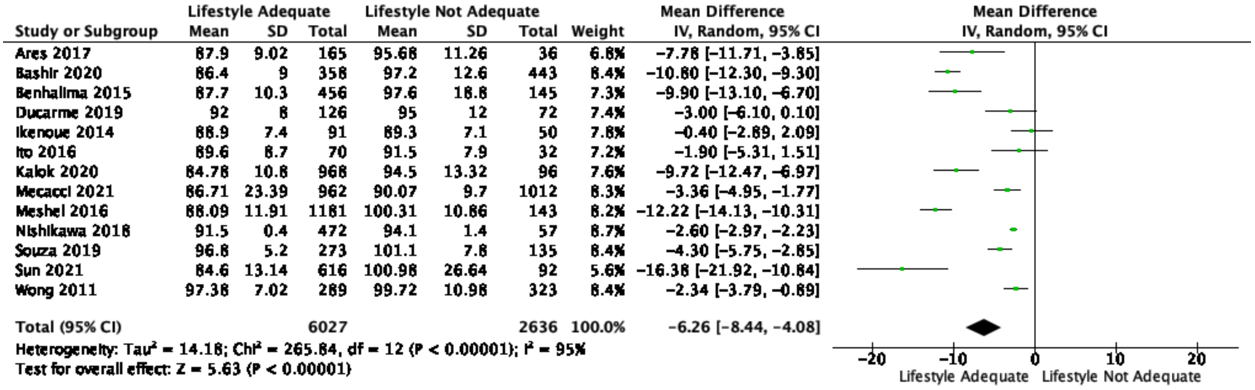

b)

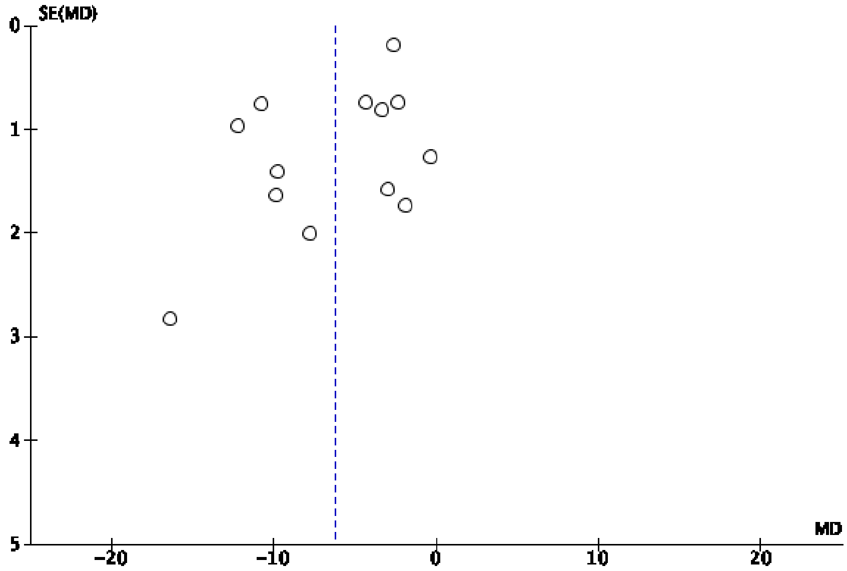

Supplementary Figure 7 Comparison of studies of whether lifestyle was adequate or inadequate according to 1-hour glucose in mg/dL

Legend. Forest plot (a) and Funnel plot (b) for included studies comparing if lifestyle was adequate (no need for escalation to pharmacological agent(s)) or not adequate (required escalation to pharmacological agent(s)).

a)

	Lifestyle Adequate			Lifestyle Not Adequate				Mean Difference
Study or Subgroup	Mean	SD	Total	Mean	SD	Total	Weight	IV, Random, 95% CI
Ares 2017	195.16	28	165	202	42.85	36	6.4%	−6.84 [−21.47, 7.79]
Bashir 2020	178.2	30.6	358	196.2	28.8	443	11.2%	−18.00 [−22.15, −13.85]
Benhalima 2015	184.5	25.8	456	194.7	30.1	145	10.7%	−10.20 [−15.64, −4.76]
Ducarme 2019	167	0.26	126	182	0.31	72	11.9%	−15.00 [−15.08, −14.92]
Ikenoue 2014	153.7	27.3	91	152.8	29	50	8.6%	0.90 [−8.90, 10.70]
Ito 2016	155.3	33.6	70	179.9	34.9	32	6.5%	−24.60 [−39.03, −10.17]
Mecacci 2021	171.48	21.12	962	177.71	21.16	1012	11.8%	−6.23 [−8.10, −4.36]
Meshel 2016	88.09	11.91	1181	100.31	10.86	143	11.7%	−12.22 [−14.13, −10.31]
Nishikawa 2018	155.4	1.5	472	184.9	4.4	57	11.9%	−29.50 [−30.65, −28.35]
Sun 2021	173.52	29.34	616	203.76	39.6	92	9.3%	−30.24 [−38.66, −21.82]
Total (95% CI)			4497			2082	100.0%	−15.33 [−20.81, −9.85]

Heterogeneity: $Tau^2 = 65.65$; $Chi^2 = 732.47$, df = 9 ($P < 0.00001$); $I^2 = 99\%$
Test for overall effect: Z = 5.48 ($P < 0.00001$)

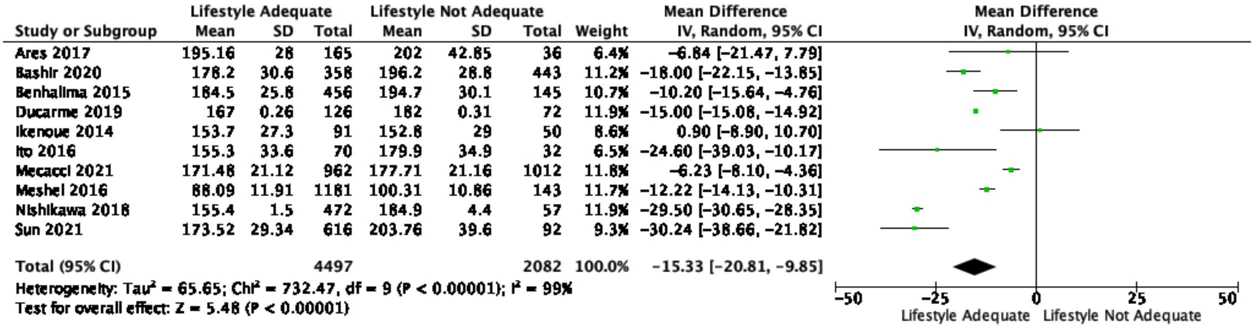

b)

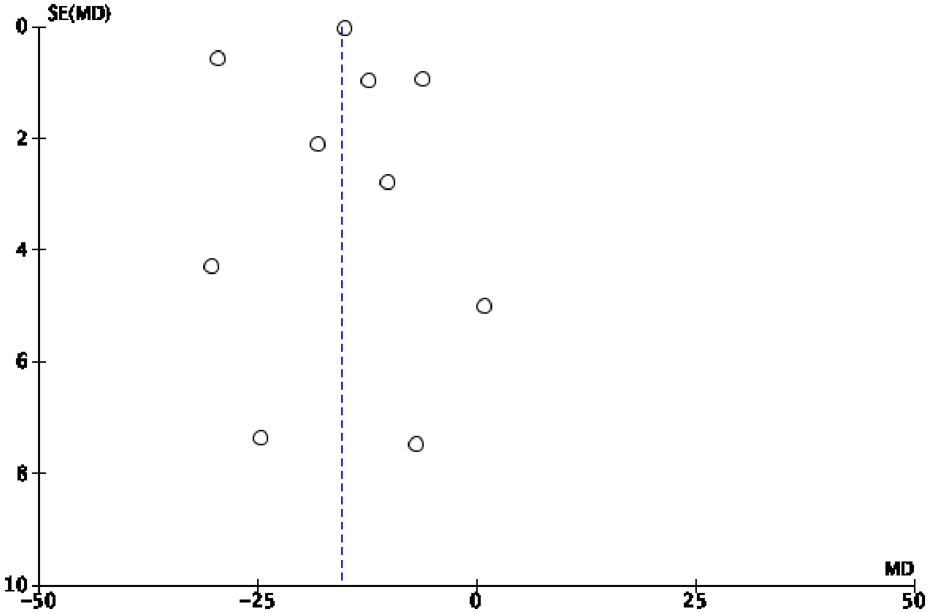

Supplementary Figure 8 Comparison of studies of whether lifestyle was adequate or inadequate according to 2-hour glucose in mg/dL

Legend. Forest plot (a) and Funnel plot (b) for included studies comparing if lifestyle was adequate (no need for escalation to pharmacological agent(s)) or not adequate (required escalation to pharmacological agent(s)).

a)

	Lifestyle Adequate			Lifestyle Not Adequate				Mean Difference
Study or Subgroup	Mean	SD	Total	Mean	SD	Total	Weight	IV, Random, 95% CI
Ares 2017	179.53	22.4	165	190.78	47.33	36	4.5%	−11.25 [−27.08, 4.58]
Bashir 2020	149.4	28.8	358	165.6	84.6	443	7.6%	−16.20 [−24.62, −7.78]
Benhalima 2015	175	22.8	456	185.2	28.5	145	9.2%	−10.20 [−15.29, −5.11]
Ducarme 2019	151	0.25	126	157	0.32	72	10.4%	−6.00 [−6.09, −5.91]
Ikenoue 2014	135.8	26.2	91	146.6	29.6	50	6.9%	−10.80 [−20.61, −0.99]
Ito 2016	135.3	29.7	70	150.5	28.5	32	5.9%	−15.20 [−27.28, −3.12]
Kalok 2020	152.82	14.58	968	156.24	18	96	9.7%	−3.42 [−7.14, 0.30]
Mecacci 2021	138.06	30.08	962	140.45	33.29	1012	10.0%	−2.39 [−5.19, 0.41]
Meshel 2016	162.22	29.68	1181	161.69	36.31	143	8.7%	0.53 [−5.66, 6.72]
Nishikawa 2018	139.5	1.2	472	157.1	4.1	57	10.3%	−17.60 [−18.67, −16.53]
Sun 2021	149.22	27.9	616	172.98	44.82	92	7.1%	−23.76 [−33.18, −14.34]
Wong 2011	149.4	22.68	289	150.12	26.64	323	9.6%	−0.72 [−4.63, 3.19]
Total (95% CI)			5754			2501	100.0%	−9.06 [−13.55, −4.56]

Heterogeneity: $Tau^2 = 50.61$; $Chi^2 = 494.03$, df = 11 ($P < 0.00001$); $I^2 = 98\%$
Test for overall effect: Z = 3.95 ($P < 0.0001$)

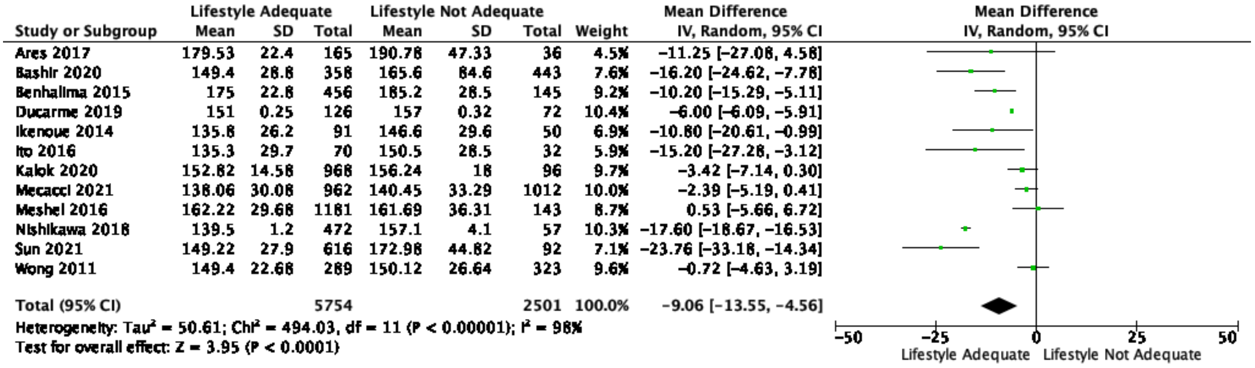

b)

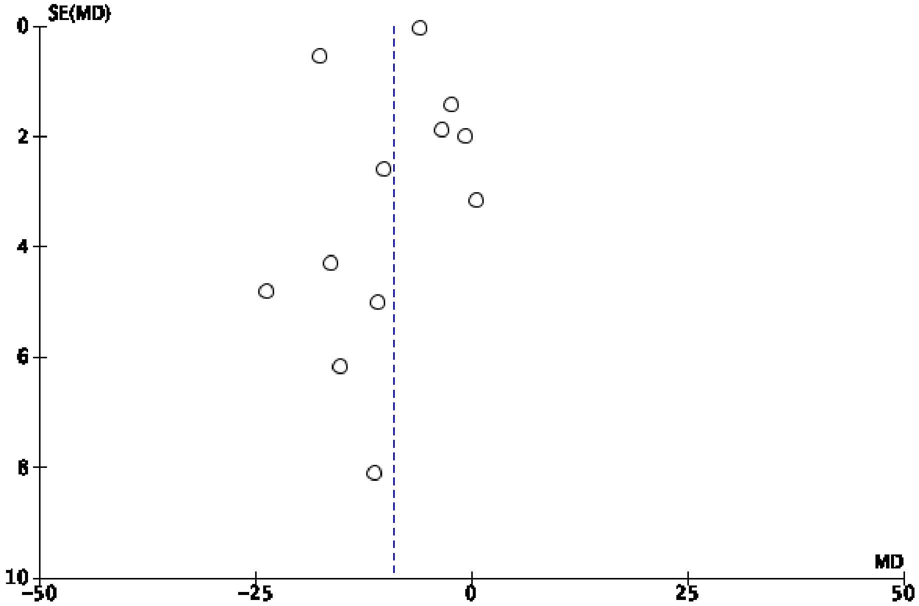

Supplementary Figure 9 Comparison of studies of whether lifestyle was adequate or inadequate according to 3-hour glucose in mg/dL

Legend. Forest plot (a) and Funnel plot (b) for included studies comparing if lifestyle was adequate (no need for escalation to pharmacological agent(s)) or not adequate (required escalation to pharmacological agent(s)).

a)

	Lifestyle Adequate			Lifestyle Not Adequate				Mean Difference
Study or Subgroup	Mean	SD	Total	Mean	SD	Total	Weight	IV, Random, 95% CI
Ares 2017	152.77	25.72	165	161.47	31.52	36	13.3%	−8.70 [−19.72, 2.32]
Benhalima 2015	145.4	27.6	456	152.7	33.4	145	45.0%	−7.30 [−13.30, −1.30]
Meshel 2016	97.87	35.49	1181	107.74	35.94	143	41.7%	−9.87 [−16.10, −3.64]
Total (95% CI)			1802			324	100.0%	−8.56 [−12.58, −4.54]

Heterogeneity: $Tau^2 = 0.00$; $Chi^2 = 0.34$, $df = 2$ ($P = 0.84$); $I^2 = 0\%$
Test for overall effect: $Z = 4.17$ ($P < 0.0001$)

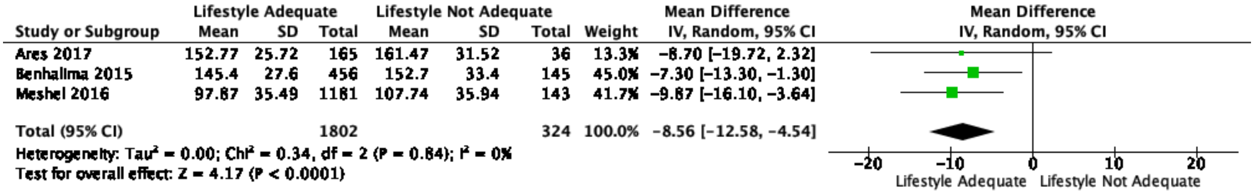

b)

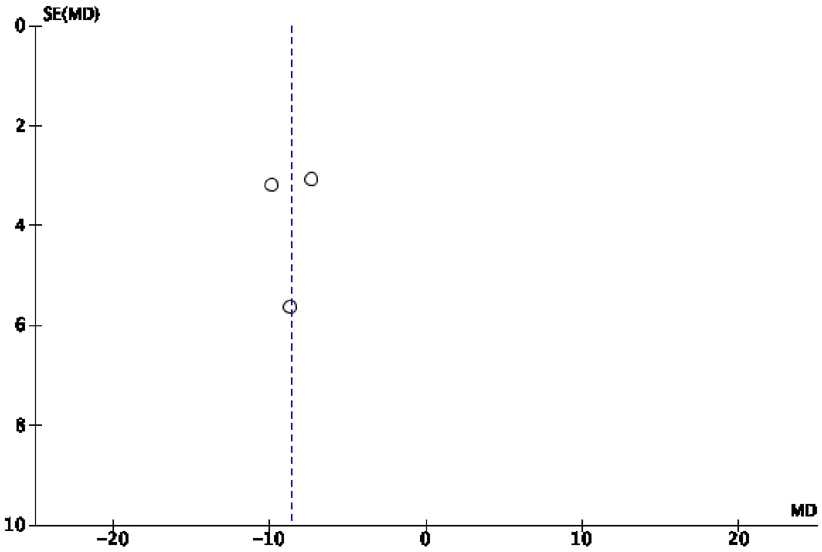

Supplementary Figure 10 Comparison of studies of whether lifestyle was adequate or inadequate according to family history of diabetes

Legend. Forest plot (a) and Funnel plot (b) for included studies comparing if lifestyle was adequate (no need for escalation to pharmacological agent(s)) or not adequate (required escalation to pharmacological agent(s)).

a)

Study or Subgroup	Lifestyle Adequate Events	Lifestyle Adequate Total	Lifestyle Not Adequate Events	Lifestyle Not Adequate Total	Weight	Odds Ratio M-H, Random, 95% CI
Benhalima 2015	75	456	33	135	6.1%	0.61 [0.38, 0.97]
Gilbert 2021	97	178	96	163	6.9%	0.84 [0.54, 1.28]
Ikenoue 2014	12	91	10	50	1.7%	0.61 [0.24, 1.53]
Ito 2016	16	70	15	32	1.9%	0.34 [0.14, 0.82]
Kalok 2020	210	968	30	96	6.2%	0.61 [0.39, 0.96]
Koning 2016	156	460	170	360	13.0%	0.57 [0.43, 0.76]
Krispin 2021	95	314	123	328	10.5%	0.72 [0.52, 1.00]
Mecacci 2021	223	962	309	1012	19.6%	0.69 [0.56, 0.84]
Meshel 2016	479	1181	66	143	9.6%	0.80 [0.56, 1.13]
Nishikawa 2018	199	472	38	57	4.1%	0.36 [0.20, 0.65]
Souza 2019	159	273	98	135	6.5%	0.53 [0.34, 0.82]
Sun 2021	46	616	10	92	2.8%	0.66 [0.32, 1.36]
Wong 2011	134	289	157	323	11.1%	0.91 [0.67, 1.26]
Total (95% CI)		6330		2926	100.0%	0.66 [0.59, 0.75]
Total events	1901		1155			

Heterogeneity: $Tau^2 = 0.01$; $Chi^2 = 15.07$, $df = 12$ ($P = 0.24$); $I^2 = 20\%$

Test for overall effect: $Z = 6.45$ ($P < 0.00001$)

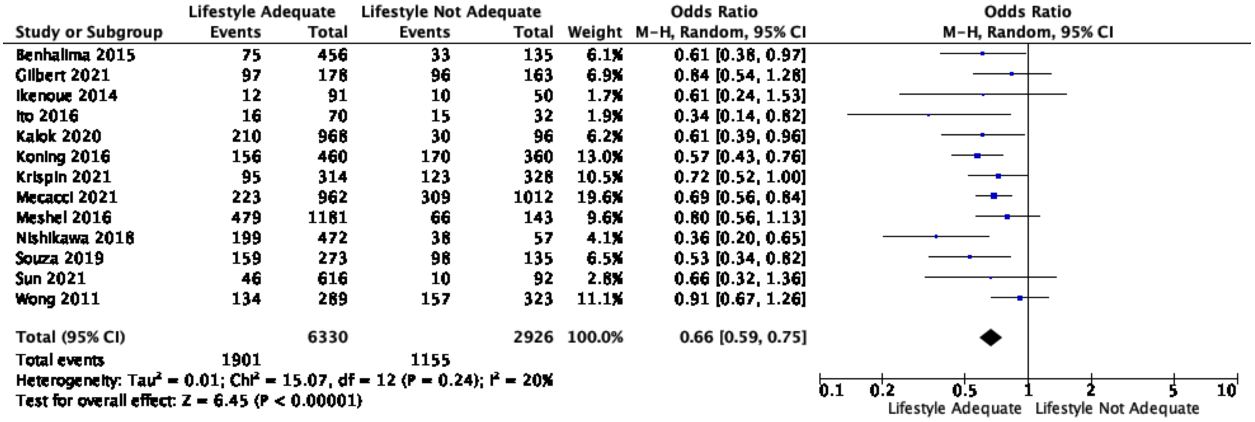

b)

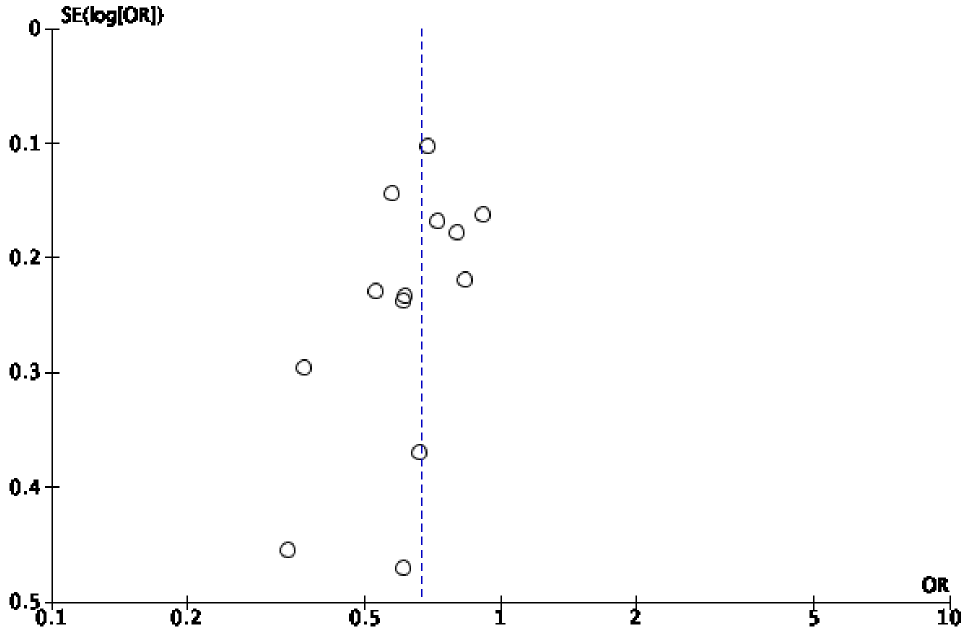

Supplementary Figure 11 Comparison of studies of whether lifestyle was adequate or inadequate according to gestational age at gestational diabetes diagnosis in weeks

Legend. Forest plot (a) and Funnel plot (b) for included studies comparing if lifestyle was adequate (no need for escalation to pharmacological agent(s)) or not adequate (required escalation to pharmacological agent(s)).

a)

	Lifestyle Adequate			Lifestyle Not Adequate				Mean Difference
Study or Subgroup	Mean	SD	Total	Mean	SD	Total	Weight	IV, Random, 95% CI
Bashir 2020	26.6	5.6	358	22.4	7.04	443	13.6%	4.20 [3.32, 5.08]
Benhalima 2015	27.1	3.7	456	25.3	4.9	145	13.6%	1.80 [0.93, 2.67]
Ikenoue 2014	22	8	91	20	8	50	5.0%	2.00 [−0.76, 4.76]
Ito 2016	20.8	7.5	70	18.6	8.4	32	3.6%	2.20 [−1.20, 5.60]
Kalok 2020	25	6	968	21.1	5.7	96	11.6%	3.90 [2.70, 5.10]
Meshel 2016	28.01	6.46	1181	22.58	8.04	143	10.6%	5.43 [4.06, 6.80]
Nishikawa 2018	25.32	0.35	472	22.18	1.1	57	16.4%	3.14 [2.85, 3.43]
Sun 2021	25.8	2.9	616	24.3	4.8	92	12.8%	1.50 [0.49, 2.51]
Wong 2011	25.5	5.5	289	22.9	7	323	12.9%	2.60 [1.61, 3.59]
Total (95% CI)			4501			1381	100.0%	3.06 [2.33, 3.79]

Heterogeneity: $Tau^2 = 0.83$; $Chi^2 = 39.15$, df = 8 ($P < 0.00001$); $I^2 = 80\%$
Test for overall effect: Z = 8.20 ($P < 0.00001$)

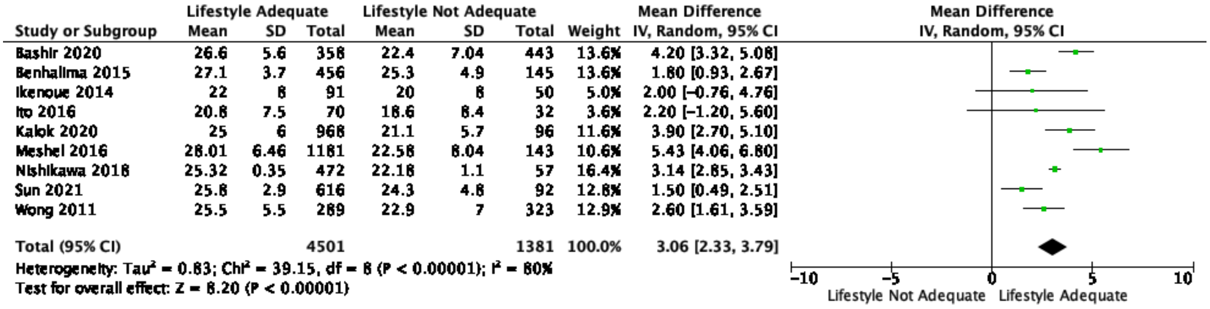

b)

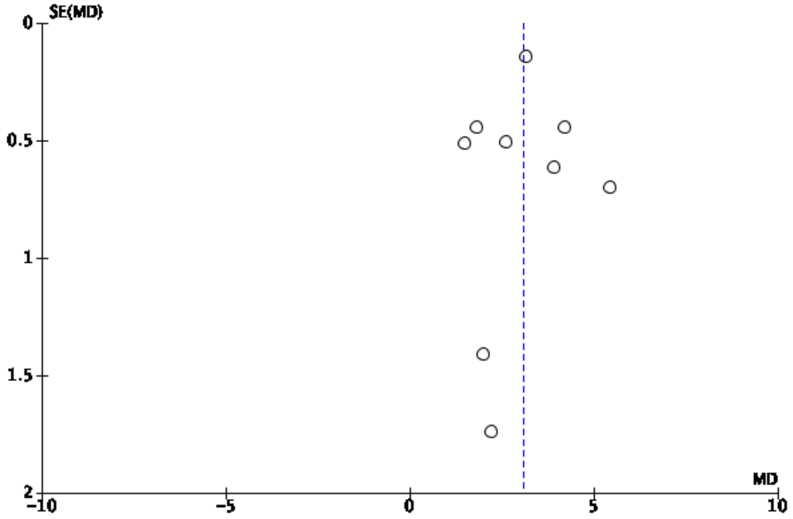

Supplementary Figure 12 Comparison of studies of whether lifestyle was adequate or inadequate according to history of smoking

Legend. Forest plot (a) and Funnel plot (b) for included studies comparing if lifestyle was adequate (no need for escalation to pharmacological agent(s)) or not adequate (required escalation to pharmacological agent(s)).

a)

	Lifestyle Adequate		Lifestyle Not Adequate			Odds Ratio
Study or Subgroup	Events	Total	Events	Total	Weight	M-H, Random, 95% CI
Durnwald 2011	30	424	6	36	13.1%	0.38 [0.15, 0.99]
Krispin 2021	22	314	29	328	22.3%	0.78 [0.44, 1.38]
Meghelli 2020	10	58	12	63	13.5%	0.89 [0.35, 2.24]
Ng 2020	218	1281	81	576	32.6%	1.25 [0.95, 1.65]
Souza 2019	19	273	15	135	18.4%	0.60 [0.29, 1.22]
Total (95% CI)		2350		1138	100.0%	0.80 [0.52, 1.23]
Total events	299		143			

Heterogeneity: $Tau^2 = 0.12$; $Chi^2 = 9.35$, df = 4 (P = 0.05); $I^2 = 57\%$
Test for overall effect: Z = 1.01 (P = 0.31)

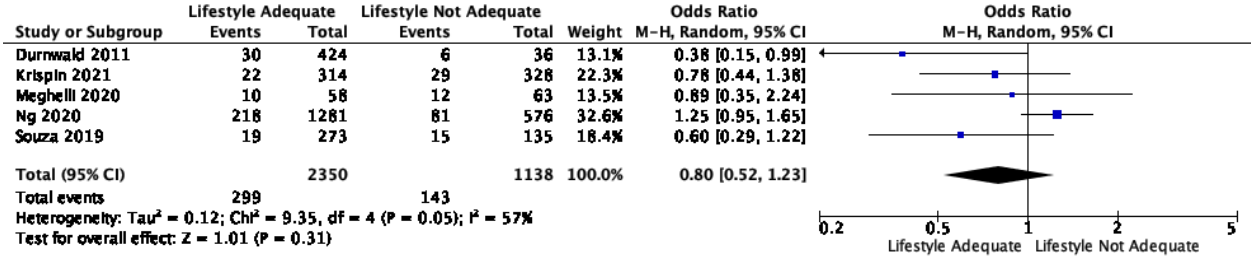

b)

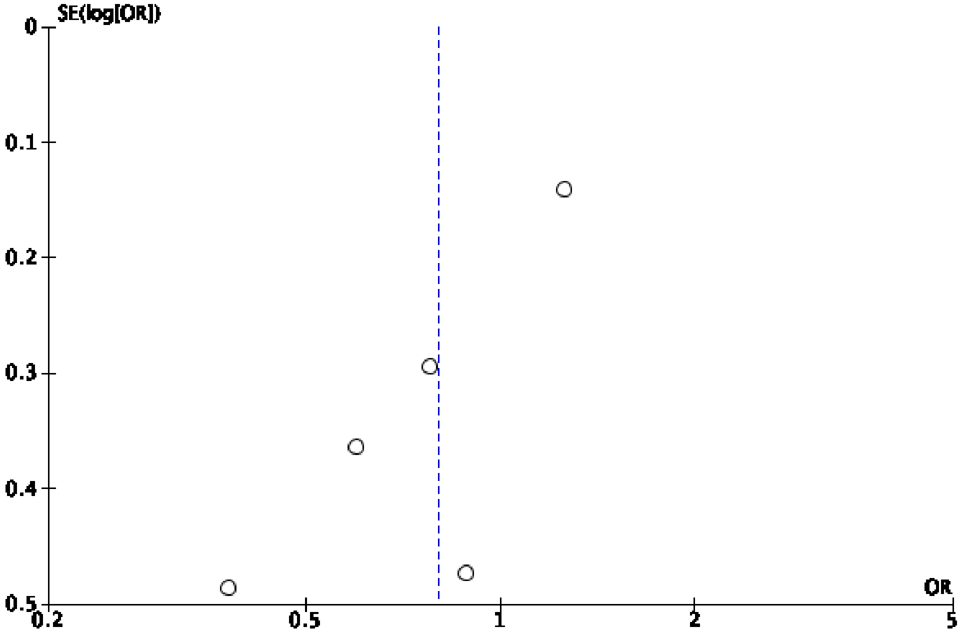

Supplementary Figure 13 Comparison of studies of whether lifestyle was adequate or inadequate according to previous history of macrosomia

Legend. Forest plot (a) and Funnel plot (b) for included studies comparing if lifestyle was adequate (no need for escalation to pharmacological agent(s)) or not adequate (required escalation to pharmacological agent(s)).

a)

	Lifestyle Adequate		Lifestyle Not Adequate			Odds Ratio
Study or Subgroup	Events	Total	Events	Total	Weight	M-H, Random, 95% CI
Ducarme 2019	7	126	8	72	9.7%	0.47 [0.16, 1.36]
Ito 2016	0	70	1	32	1.5%	0.15 [0.01, 3.76]
Koning 2016	55	460	35	360	20.6%	1.26 [0.81, 1.97]
Krispin 2021	19	314	29	328	17.2%	0.66 [0.36, 1.21]
Mecacci 2021	11	962	14	1012	13.5%	0.82 [0.37, 1.83]
Ouzounian 2011	116	1169	49	262	22.4%	0.47 [0.33, 0.68]
Souza 2019	16	273	19	135	15.2%	0.38 [0.19, 0.77]
Total (95% CI)		3394		2201	100.0%	0.63 [0.42, 0.94]
Total events	224		155			

Heterogeneity: $Tau^2 = 0.16$; $Chi^2 = 15.23$, df = 6 (P = 0.02); $I^2 = 61\%$

Test for overall effect: Z = 2.23 (P = 0.03)

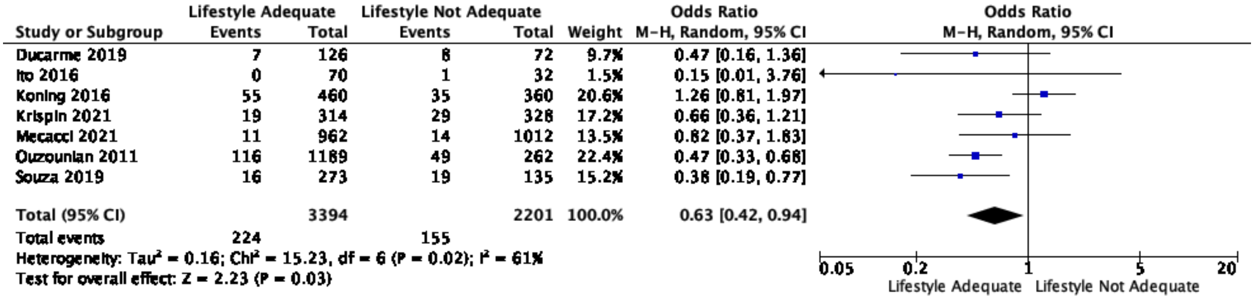

b)

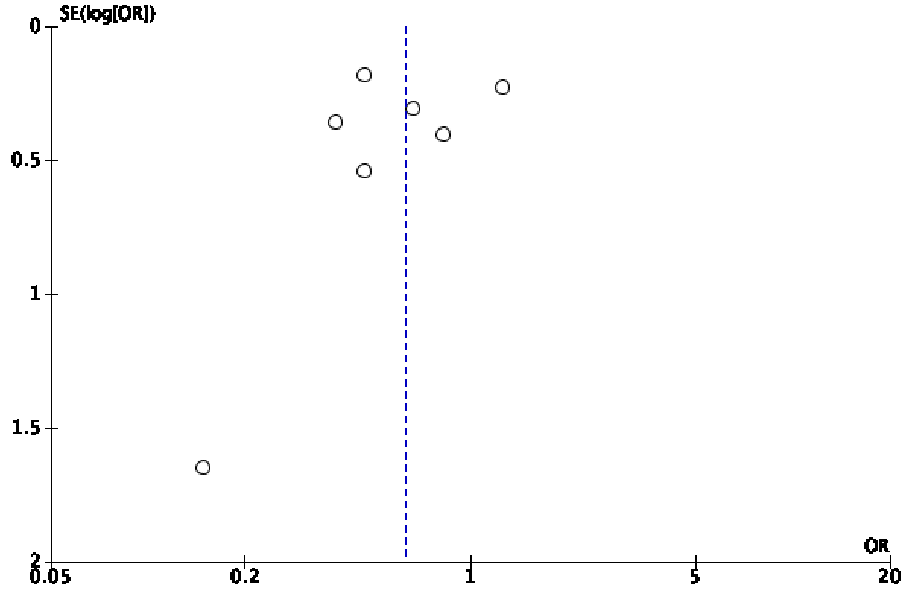

Supplementary Figure 14 Comparison of studies of whether oral pharmacological agent was adequate in controlling glucose or inadequate according to maternal age in years

Legend. Forest plot (a) and Funnel plot (b) for included studies comparing if lifestyle was adequate (no need for escalation to insulin) or not adequate (required escalation to insulin).

a)

Study or Subgroup	Oral agent success Mean	SD	Total	Oral agent failure Mean	SD	Total	Weight	Mean Difference IV, Random, 95% CI
Chmait 2004	32	5	56	31	7	13	4.8%	1.00 [−3.02, 5.02]
Conway 2004	31.3	6.4	63	30.3	5	12	6.4%	1.00 [−2.24, 4.24]
Gante 2018	32.9	5.4	253	35.2	5	135	13.6%	−2.30 [−3.37, −1.23]
Harper 2016	29.7	5.8	157	30.1	5.1	63	11.8%	−0.40 [−1.95, 1.15]
Kahn 2006	29	5	77	34	5	18	8.2%	−5.00 [−7.57, −2.43]
Khin 2018	30	5.9	61	32.4	5.5	77	10.4%	−2.40 [−4.32, −0.48]
McGrath 2016	32.9	4.6	29	32.8	5.3	34	8.6%	0.10 [−2.34, 2.54]
Picón-César 2021	34.84	5.75	70	33.75	3.85	20	9.6%	1.09 [−1.07, 3.25]
Rochon 2006	30.5	5.8	80	31.3	6.2	21	7.2%	−0.80 [−3.74, 2.14]
Tertti 2013	31.4	4	87	33.8	4.9	23	9.5%	−2.40 [−4.57, −0.23]
Yogev 2011	33.2	5	93	32.7	5.3	31	9.7%	0.50 [−1.62, 2.62]
Total (95% CI)			1026			447	100.0%	−1.04 [−2.10, 0.03]

Heterogeneity: $Tau^2 = 1.86$; $Chi^2 = 26.88$, df = 10 (P = 0.003); $I^2 = 63\%$
Test for overall effect: Z = 1.91 (P = 0.06)

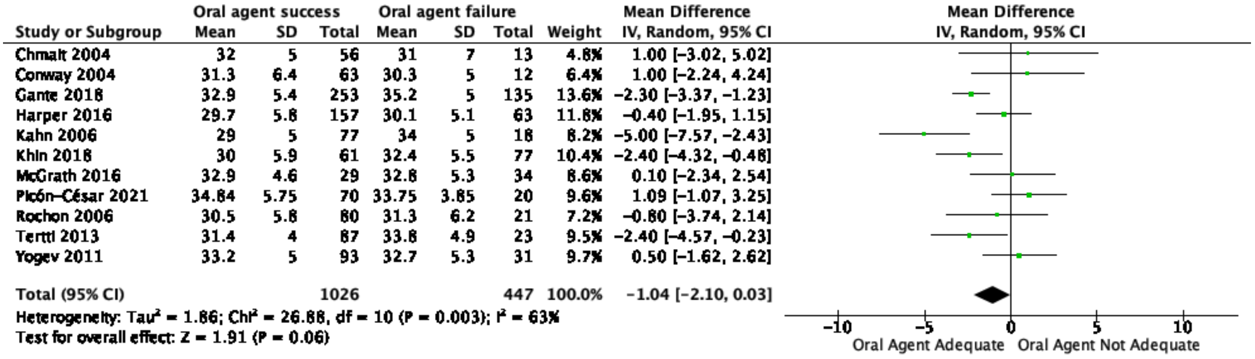

b)

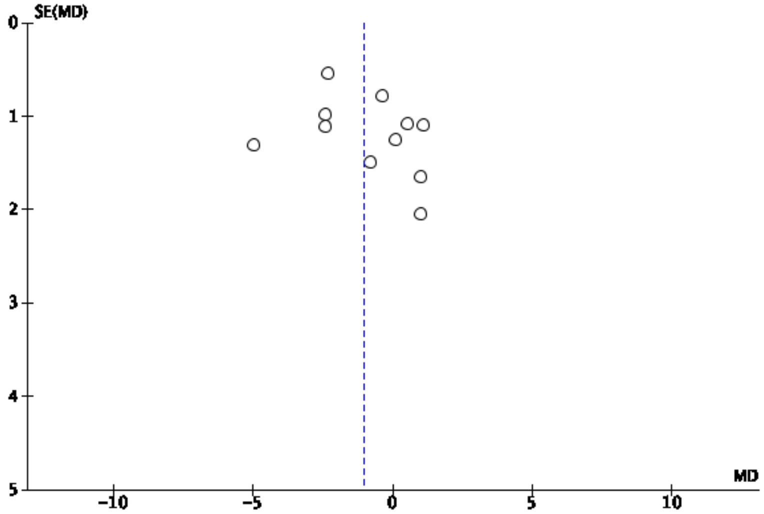

Supplementary Figure 15 Comparison of studies of whether oral pharmacological agent was adequate in controlling glucose or inadequate according to nulliparity

Legend. Forest plot (a) and Funnel plot (b) for included studies comparing if lifestyle was adequate (no need for escalation to insulin) or not adequate (required escalation to insulin).

a)

	Oral agent success		Oral agent failure			Odds Ratio
Study or Subgroup	Events	Total	Events	Total	Weight	M-H, Random, 95% CI
Chmait 2004	8	56	1	13	1.6%	2.00 [0.23, 17.57]
Harper 2016	47	157	14	63	16.5%	1.50 [0.75, 2.97]
Khin 2018	21	61	23	77	14.9%	1.23 [0.60, 2.53]
Picón-César 2021	44	70	12	20	7.5%	1.13 [0.41, 3.12]
Rochon 2006	24	80	3	21	4.5%	2.57 [0.69, 9.55]
Rowan 2008	75	195	40	168	36.9%	2.00 [1.27, 3.16]
Tertti 2013	34	87	8	23	8.4%	1.20 [0.46, 3.14]
Yogev 2011	28	93	9	31	9.7%	1.05 [0.43, 2.57]
Total (95% CI)		799		416	100.0%	1.55 [1.17, 2.04]
Total events	281		110			

Heterogeneity: $Tau^2 = 0.00$; $Chi^2 = 3.58$, df = 7 (P = 0.83); $I^2 = 0\%$
Test for overall effect: Z = 3.08 (P = 0.002)

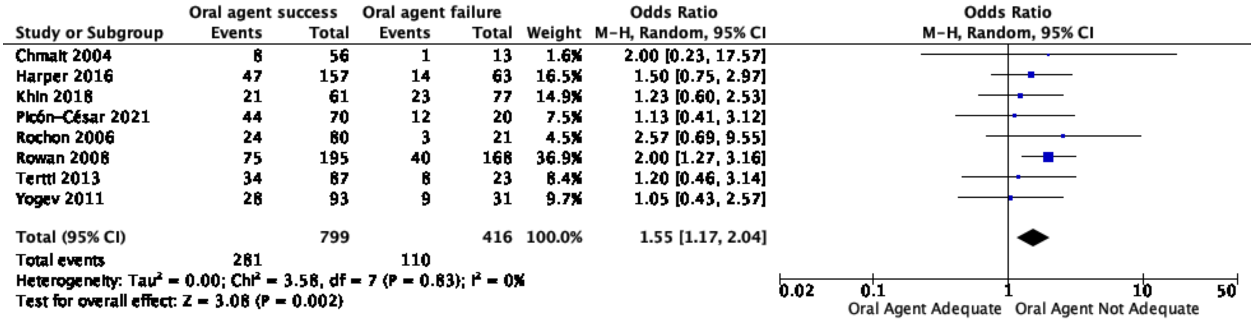

b)

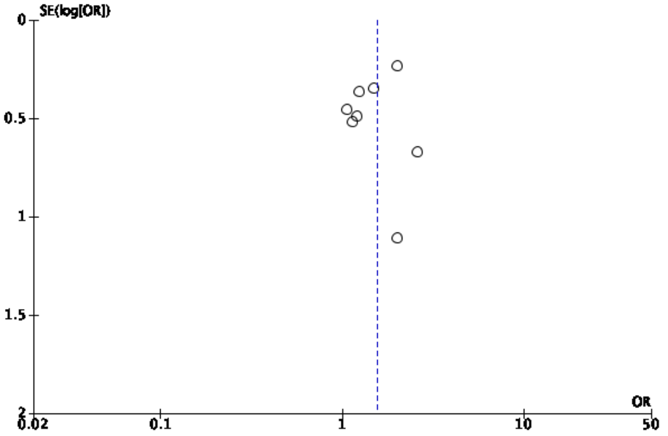

Supplementary Figure 16 Comparison of studies of whether oral pharmacological agent was adequate in controlling glucose or inadequate according to body mass index kg/m^2

Legend. Forest plot (a) and Funnel plot (b) for included studies comparing if lifestyle was adequate (no need for escalation to insulin) or not adequate (required escalation to insulin).

a)

	Oral agent success			Oral agent failure				Mean Difference
Study or Subgroup	Mean	SD	Total	Mean	SD	Total	Weight	IV, Random, 95% CI
Gante 2018	29	6.3	253	31.8	6.6	135	17.0%	−2.80 [−4.16, −1.44]
Harper 2016	35	8.6	157	35.4	8.2	63	10.0%	−0.40 [−2.83, 2.03]
Kahn 2006	30	6	77	32	8	18	5.1%	−2.00 [−5.93, 1.93]
Khin 2018	32.4	7.1	61	32.7	7.1	77	10.3%	−0.30 [−2.69, 2.09]
McGrath 2016	25.9	8.3	29	30	7.8	34	5.0%	−4.10 [−8.10, −0.10]
Picón–César 2021	29.75	6.26	70	30.65	4.58	20	9.8%	−0.90 [−3.39, 1.59]
Rochon 2006	31.5	7.5	80	32.2	5	21	8.8%	−0.70 [−3.40, 2.00]
Rowan 2008	31.1	7.8	195	33.6	8.6	168	14.4%	−2.50 [−4.20, −0.80]
Tertti 2013	29.8	6	87	28.2	5.8	23	8.9%	1.60 [−1.08, 4.28]
Yogev 2011	28	5.4	93	27.9	5.8	31	10.6%	0.10 [−2.22, 2.42]
Total (95% CI)			1102			590	100.0%	−1.21 [−2.21, −0.21]

Heterogeneity: $Tau^2 = 1.05$; $Chi^2 = 15.80$, df = 9 (P = 0.07); $I^2 = 43\%$
Test for overall effect: Z = 2.38 (P = 0.02)

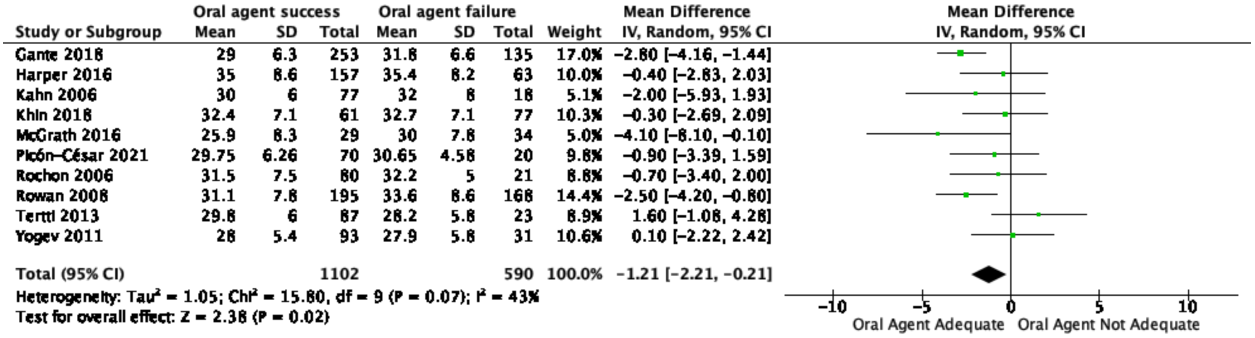

b)

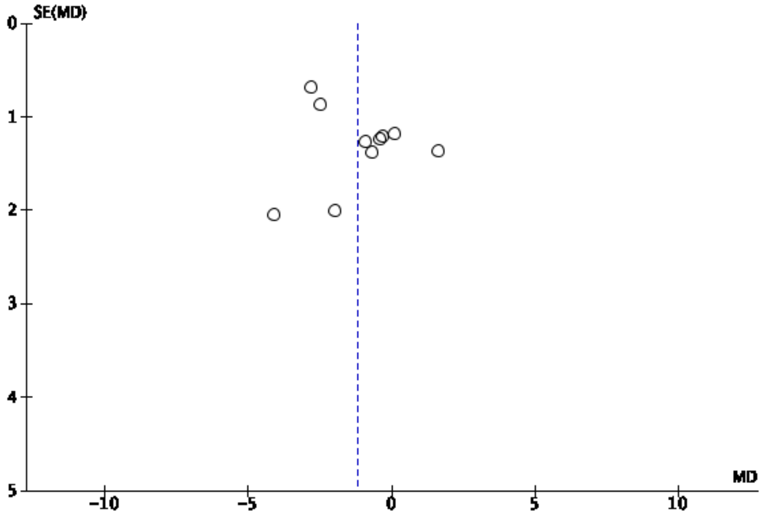

Supplementary Figure 17 Comparison of studies of whether oral pharmacological agent was adequate in controlling glucose or inadequate according to previous history of gestational diabetes

Legend. Forest plot (a) and Funnel plot (b) for included studies comparing if lifestyle was adequate (no need for escalation to insulin) or not adequate (required escalation to insulin).

a)

	Oral agent success		Oral agent failure			Odds Ratio
Study or Subgroup	Events	Total	Events	Total	Weight	M-H, Random, 95% CI
Chmait 2004	9	56	4	13	6.2%	0.43 [0.11, 1.71]
Gante 2018	25	253	21	135	18.3%	0.60 [0.32, 1.11]
Harper 2016	22	157	30	63	17.0%	0.18 [0.09, 0.35]
Kahn 2006	10	77	5	18	7.5%	0.39 [0.11, 1.32]
McGrath 2016	5	29	4	34	5.9%	1.56 [0.38, 6.47]
Picón-César 2021	19	70	9	20	9.9%	0.46 [0.16, 1.27]
Rowan 2008	38	195	56	168	23.0%	0.48 [0.30, 0.78]
Yogev 2011	19	93	12	31	12.2%	0.41 [0.17, 0.98]
Total (95% CI)		930		482	100.0%	0.43 [0.30, 0.63]
Total events	147		141			

Heterogeneity: $Tau^2 = 0.10$; $Chi^2 = 11.09$, df = 7 ($P = 0.13$); $I^2 = 37\%$
Test for overall effect: Z = 4.36 ($P < 0.0001$)

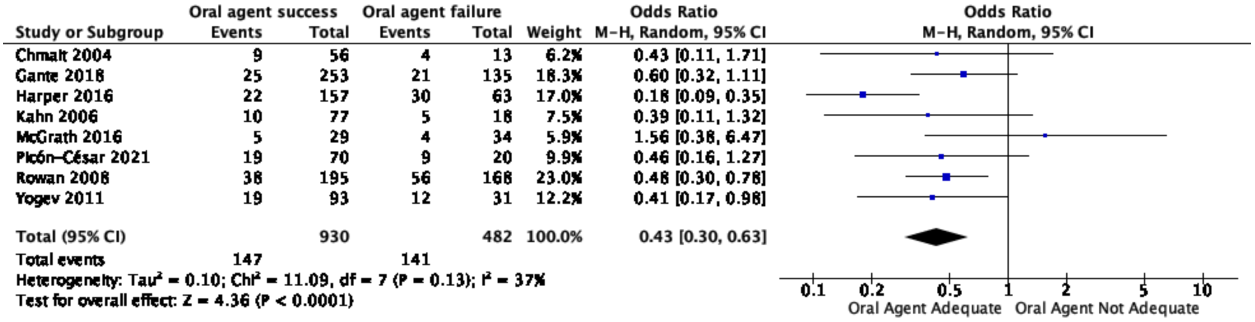

b)

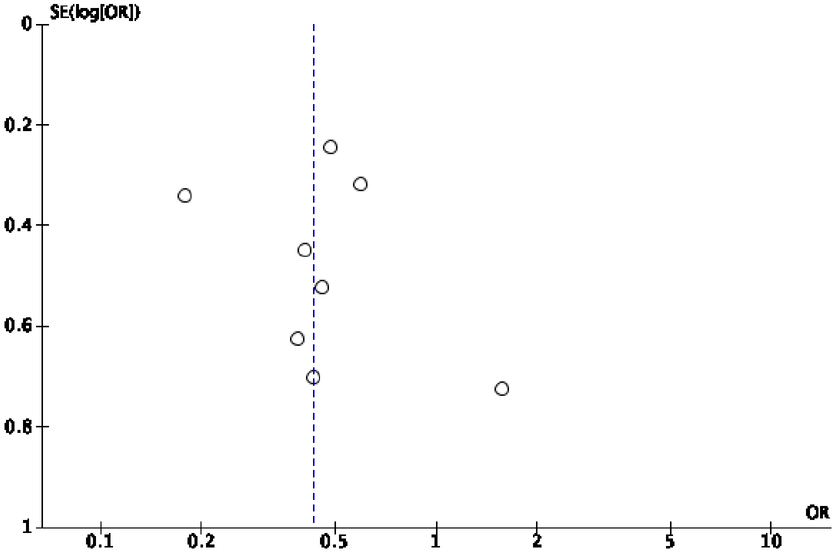

Supplementary Figure 18 Comparison of studies of whether oral pharmacological agent was adequate in controlling glucose or inadequate according to haemoglobin A1C in %

Legend. Forest plot (a) and Funnel plot (b) for included studies comparing if lifestyle was adequate (no need for escalation to insulin) or not adequate (required escalation to insulin).

a)

	Oral agent success			Oral agent failure				Mean Difference
Study or Subgroup	Mean	SD	Total	Mean	SD	Total	Weight	IV, Random, 95% CI
Gante 2018	5.2	0.5	253	5.5	0.5	135	21.8%	−0.30 [−0.40, −0.20]
Khin 2018	5.4	0.33	61	5.6	0.48	77	17.3%	−0.20 [−0.34, −0.06]
McGrath 2016	5	0.3	29	5.2	0.4	34	13.1%	−0.20 [−0.37, −0.03]
Picón–César 2021	5.3	0.37	70	5.3	0.39	20	11.5%	0.00 [−0.19, 0.19]
Rowan 2008	5.6	0.6	195	5.9	0.7	168	17.3%	−0.30 [−0.44, −0.16]
Tertti 2013	5.44	0.35	87	5.6	0.24	23	19.0%	−0.16 [−0.28, −0.04]
Total (95% CI)			695			457	100.0%	−0.21 [−0.29, −0.13]

Heterogeneity: $Tau^2 = 0.00$; $Chi^2 = 9.71$, df = 5 ($P = 0.08$); $I^2 = 49\%$
Test for overall effect: Z = 5.17 ($P < 0.00001$)

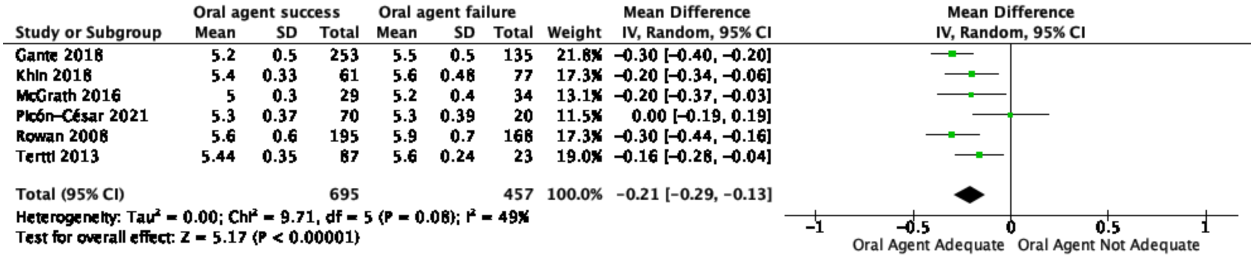

b)

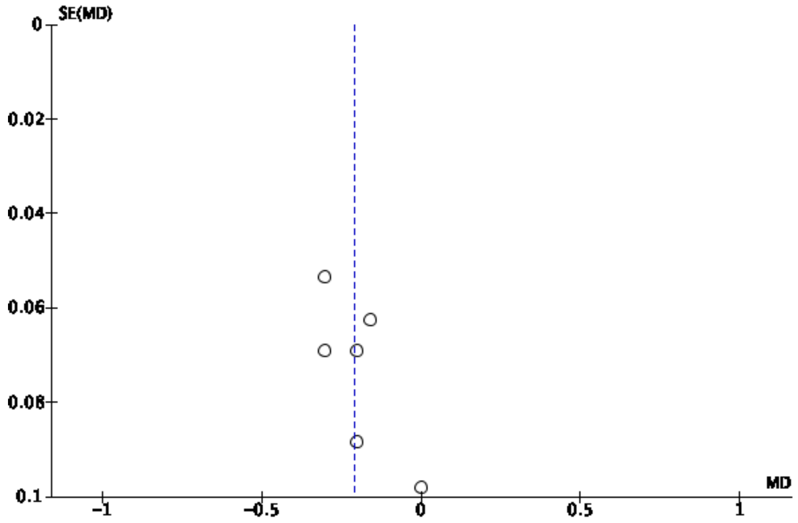

Supplementary Figure 19 Comparison of studies of whether oral pharmacological agent was adequate in controlling glucose or inadequate according to fasting glucose in mg/dL

Legend. Forest plot (a) and Funnel plot (b) for included studies comparing if lifestyle was adequate (no need for escalation to insulin) or not adequate (required escalation to insulin).

a)

	Oral agent success			Oral agent failure				Mean Difference
Study or Subgroup	Mean	SD	Total	Mean	SD	Total	Weight	IV, Random, 95% CI
Chmait 2004	94	15	56	105	22	13	5.3%	−11.00 [−23.59, 1.59]
Conway 2004	102	14	63	115	24	12	4.7%	−13.00 [−27.01, 1.01]
Gante 2018	85.4	11.41	253	92.7	15.3	135	11.2%	−7.30 [−10.24, −4.36]
Harper 2016	106	15	157	138	52	63	5.1%	−32.00 [−45.05, −18.95]
Kahn 2006	100	17	77	112	24	18	5.7%	−12.00 [−23.72, −0.28]
Khin 2018	86.9	11.9	61	97.2	16.6	77	10.2%	−10.30 [−15.06, −5.54]
McGrath 2016	86.4	10.8	29	88.2	10.8	34	9.8%	−1.80 [−7.15, 3.55]
Picón-César 2021	91.71	10.81	70	97.48	9.91	20	10.0%	−5.77 [−10.80, −0.74]
Rochon 2006	102	22	80	107	15	21	7.9%	−5.00 [−13.02, 3.02]
Rowan 2008	95.4	14.4	195	109.8	19.8	168	10.9%	−14.40 [−18.01, −10.79]
Tertti 2013	99	9	87	102.6	12.6	23	9.7%	−3.60 [−9.09, 1.89]
Yogev 2011	95.9	16.2	93	91.7	12.6	31	9.7%	4.20 [−1.32, 9.72]
Total (95% CI)			1221			615	100.0%	−8.02 [−11.87, −4.16]

Heterogeneity: $Tau^2 = 32.19$; $Chi^2 = 54.70$, $df = 11$ ($P < 0.00001$); $I^2 = 80\%$
Test for overall effect: $Z = 4.07$ ($P < 0.0001$)

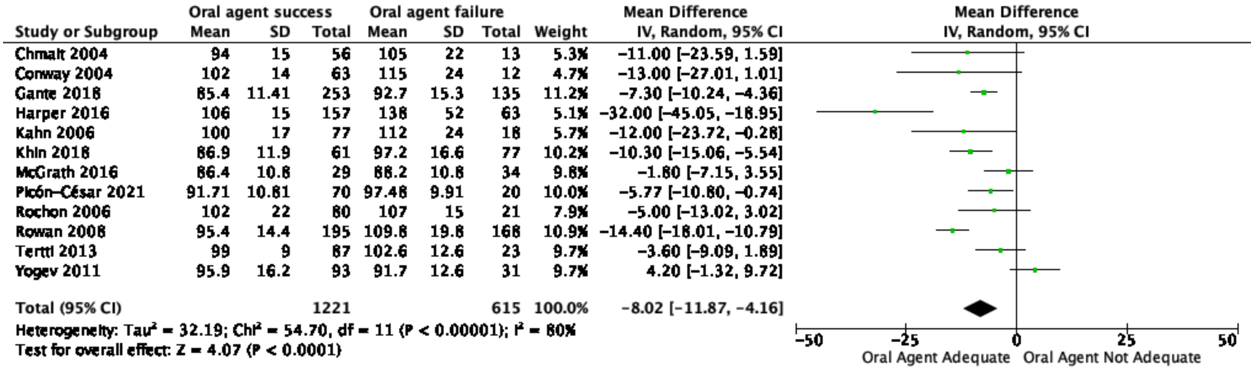

b)

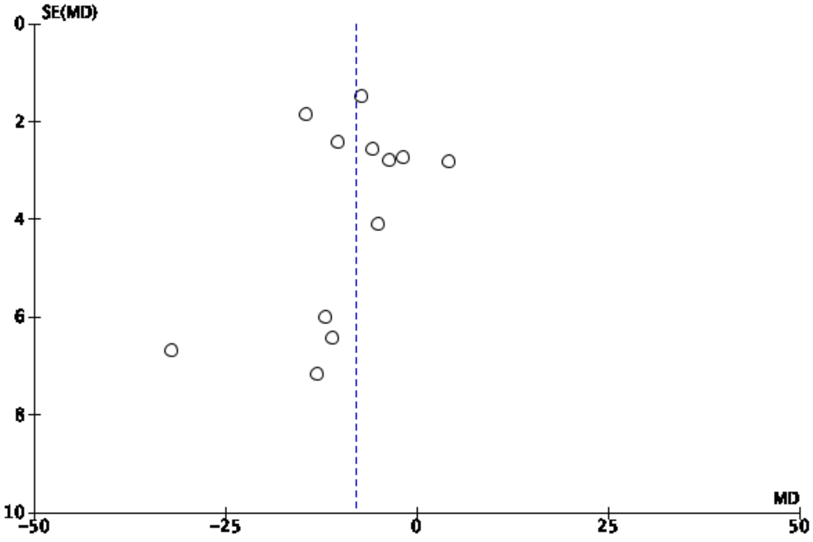

Supplementary Figure 20 Comparison of studies of whether oral pharmacological agent was adequate in controlling glucose or inadequate according to 1-hour glucose in mg/dL

Legend. Forest plot (a) and Funnel plot (b) for included studies comparing if lifestyle was adequate (no need for escalation to insulin) or not adequate (required escalation to insulin).

a)

	Oral agent success			Oral agent failure				Mean Difference
Study or Subgroup	Mean	SD	Total	Mean	SD	Total	Weight	IV, Random, 95% CI
Chmait 2004	199	25	56	206	58	13	4.4%	−7.00 [−39.20, 25.20]
Conway 2004	205	23	63	230	36	12	7.9%	−25.00 [−46.15, −3.85]
Gante 2018	171.4	32.2	253	176.8	33.1	135	17.3%	−5.40 [−12.25, 1.45]
Harper 2016	201	28	157	218	31	63	15.9%	−17.00 [−25.82, −8.18]
Picón-César 2021	198.92	30.81	70	217.31	9.64	20	16.2%	−18.39 [−26.75, −10.03]
Rochon 2006	200	33	80	223	31	21	11.2%	−23.00 [−38.10, −7.90]
Tertti 2013	201.6	25.2	87	201.6	28.8	23	12.8%	0.00 [−12.91, 12.91]
Yogev 2011	198.8	29.4	93	193	25.3	31	14.4%	5.80 [−4.92, 16.52]
Total (95% CI)			859			318	100.0%	−10.64 [−18.25, −3.02]

Heterogeneity: $Tau^2 = 75.01$; $Chi^2 = 23.47$, $df = 7$ ($P = 0.001$); $I^2 = 70\%$
Test for overall effect: $Z = 2.74$ ($P = 0.006$)

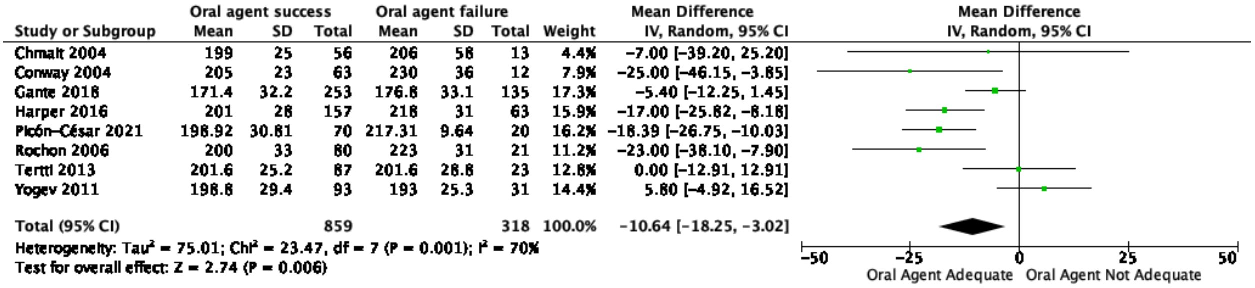

b)

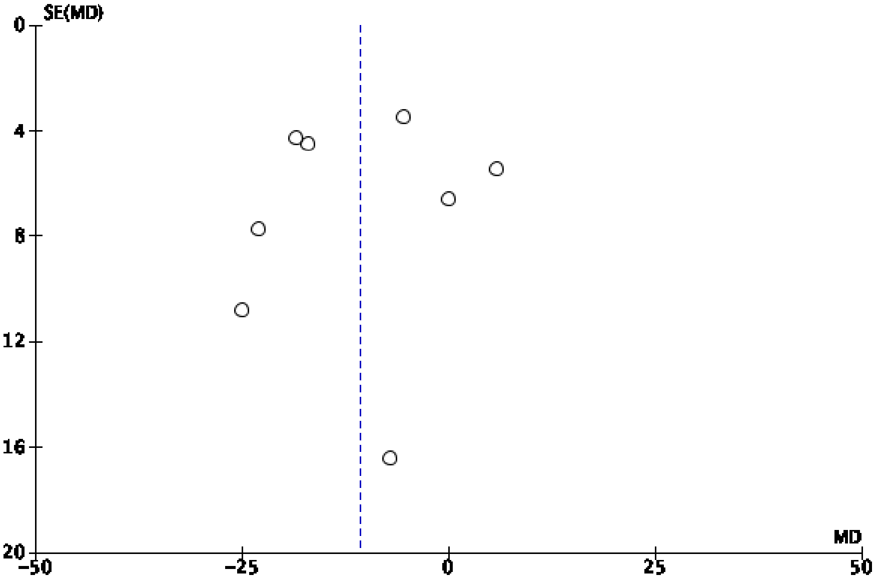

Supplementary Figure 21 Comparison of studies of whether oral pharmacological agent was adequate in controlling glucose or inadequate according to 2-hour glucose in mg/dL

Legend. Forest plot (a) and Funnel plot (b) for included studies comparing if lifestyle was adequate (no need for escalation to insulin) or not adequate (required escalation to insulin).

a)

	Oral agent success			Oral agent failure				Mean Difference
Study or Subgroup	Mean	SD	Total	Mean	SD	Total	Weight	IV, Random, 95% CI
Chmait 2004	169	31	56	192	25	13	6.2%	−23.00 [−38.83, −7.17]
Conway 2004	169	34	63	204	66	12	1.1%	−35.00 [−73.27, 3.27]
Gante 2018	144.6	33.8	253	152.9	40	135	21.2%	−8.30 [−16.23, −0.37]
Harper 2016	178	28	157	188	42	63	11.7%	−10.00 [−21.26, 1.26]
Khin 2018	154.6	21.2	61	159.7	27	77	20.8%	−5.10 [−13.14, 2.94]
McGrath 2016	153	25.2	29	149.4	25.2	34	9.7%	3.60 [−8.88, 16.08]
Picón-César 2021	185.41	25.77	70	195.14	32.25	20	6.6%	−9.73 [−25.10, 5.64]
Rochon 2006	179	33	80	189	46	21	3.6%	−10.00 [−30.96, 10.96]
Tertti 2013	147.6	32.4	87	153	28.8	23	8.3%	−5.40 [−19.00, 8.20]
Yogev 2011	165.9	31.8	93	169.6	27.8	31	10.8%	−3.70 [−15.43, 8.03]
Total (95% CI)			949			429	100.0%	−7.31 [−11.38, −3.25]

Heterogeneity: $Tau^2 = 3.88$; $Chi^2 = 9.88$, $df = 9$ ($P = 0.36$); $I^2 = 9\%$
Test for overall effect: $Z = 3.53$ ($P = 0.0004$)

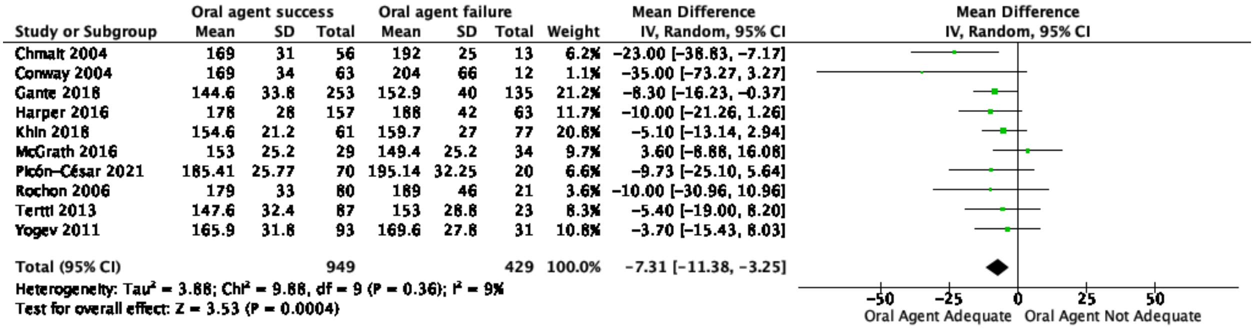

b)

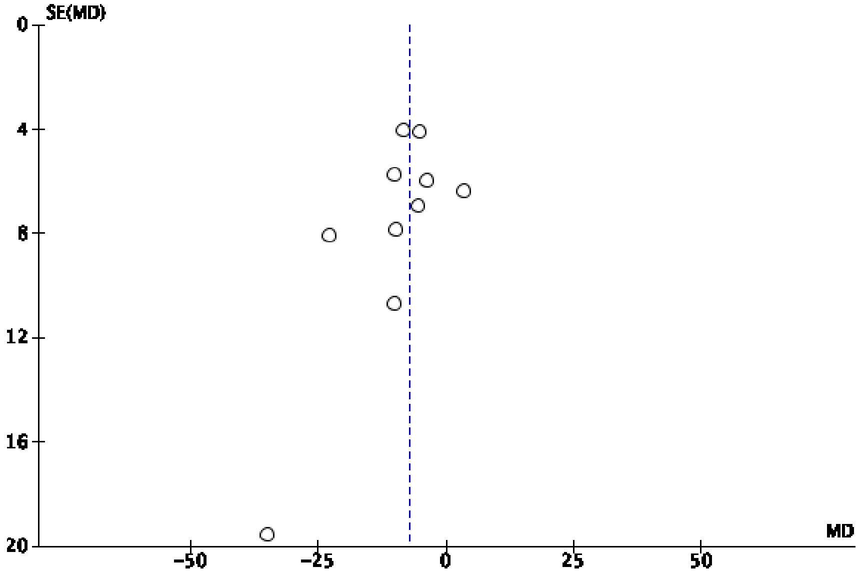

Supplementary Figure 22 Comparison of studies of whether oral pharmacological agent was adequate in controlling glucose or inadequate according to 3-hour glucose in mg/dL

Legend. Forest plot (a) and Funnel plot (b) for included studies comparing if lifestyle was adequate (no need for escalation to insulin) or not adequate (required escalation to insulin).

a)

	Oral agent success			Oral agent failure				Mean Difference
Study or Subgroup	Mean	SD	Total	Mean	SD	Total	Weight	IV, Random, 95% CI
Chmait 2004	126	33	56	128	45	13	12.3%	−2.00 [−27.94, 23.94]
Conway 2004	133	33	63	176	65	12	7.4%	−43.00 [−80.67, −5.33]
Harper 2016	140	27	157	143	42	63	23.9%	−3.00 [−14.20, 8.20]
Picón-César 2021	150.81	29.37	70	147.21	43.96	20	15.9%	3.60 [−16.86, 24.06]
Rochon 2006	138	35	80	114	38	21	17.8%	24.00 [6.03, 41.97]
Yogev 2011	118	36.3	93	121.1	28.9	31	22.6%	−3.10 [−15.67, 9.47]
Total (95% CI)			519			160	100.0%	0.00 [−11.79, 11.79]

Heterogeneity: $Tau^2 = 118.73$; $Chi^2 = 12.50$, df = 5 ($P = 0.03$); $I^2 = 60\%$
Test for overall effect: $Z = 0.00$ ($P = 1.00$)

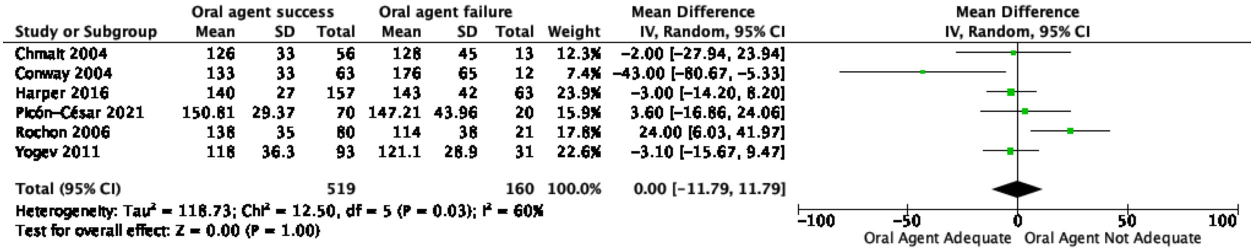

b)

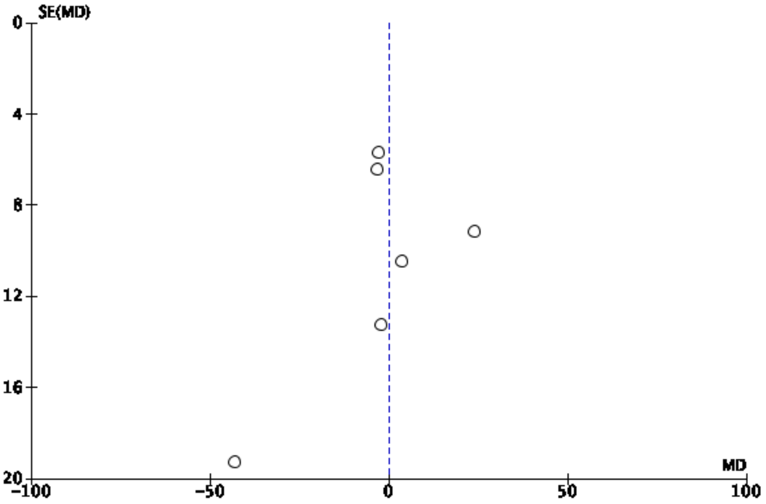

Supplementary Figure 23 Comparison of studies of whether oral pharmacological agent was adequate in controlling glucose or inadequate according to family history of diabetes

Legend. Forest plot (a) and Funnel plot (b) for included studies comparing if lifestyle was adequate (no need for escalation to insulin) or not adequate (required escalation to insulin).

a)

Study or Subgroup	Oral agent success Events	Oral agent success Total	Oral agent failure Events	Oral agent failure Total	Weight	Odds Ratio M-H, Random, 95% CI
Chmait 2004	23	50	5	11	9.4%	1.02 [0.28, 3.79]
Conway 2004	46	63	10	12	6.7%	0.54 [0.11, 2.73]
Gante 2018	114	253	53	135	29.0%	1.27 [0.83, 1.94]
McGrath 2016	14	29	15	34	13.8%	1.18 [0.44, 3.20]
Picón-César 2021	37	70	15	20	11.8%	0.37 [0.12, 1.14]
Rowan 2008	74	195	88	168	29.3%	0.56 [0.37, 0.85]
Total (95% CI)		660		380	100.0%	0.79 [0.50, 1.25]
Total events	308		186			

Heterogeneity: $Tau^2 = 0.14$; $Chi^2 = 10.15$, $df = 5$ ($P = 0.07$); $I^2 = 51\%$

Test for overall effect: $Z = 1.00$ ($P = 0.32$)

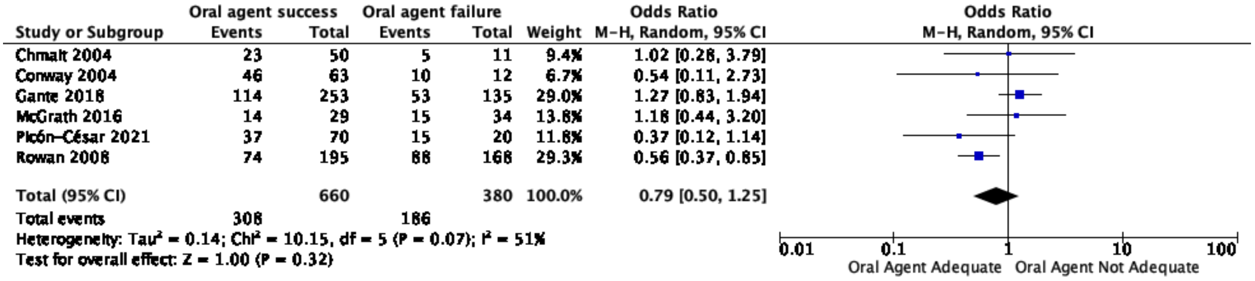

b)

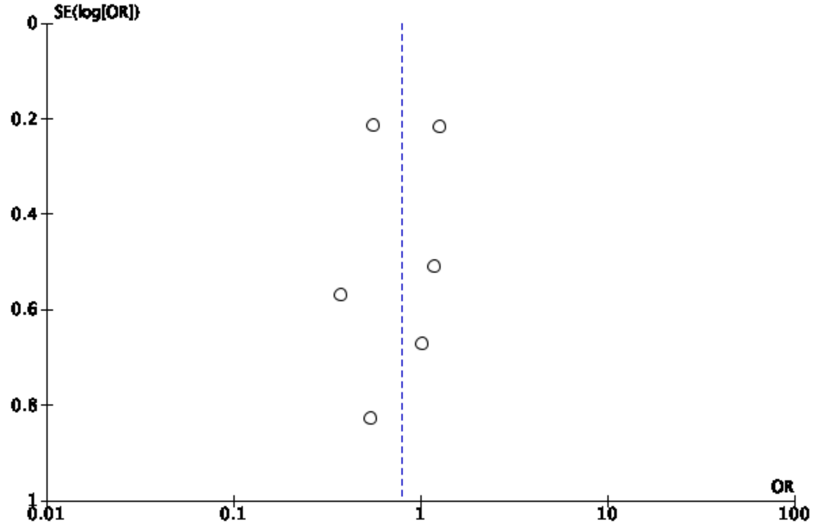

Supplementary Figure 24 Comparison of studies of whether oral pharmacological agent was adequate in controlling glucose or inadequate according to gestational age at gestational diabetes diagnosis in weeks

Legend. Forest plot (a) and Funnel plot (b) for included studies comparing if lifestyle was adequate (no need for escalation to insulin) or not adequate (required escalation to insulin).

a)

	Oral agent success			Oral agent failure				Mean Difference
Study or Subgroup	Mean	SD	Total	Mean	SD	Total	Weight	IV, Random, 95% CI
Chmait 2004	27.3	7.5	56	20	8.6	13	4.3%	7.30 [2.23, 12.37]
Conway 2004	20	8.3	63	18.4	8.4	12	4.2%	1.60 [−3.58, 6.78]
Gante 2018	20.3	8.1	253	17.5	8	135	12.6%	2.80 [1.12, 4.48]
Harper 2016	25.7	3.7	157	19.6	7.8	63	11.4%	6.10 [4.09, 8.11]
Kahn 2006	28	5	77	22.7	7	18	7.2%	5.30 [1.88, 8.72]
Khin 2018	27	5	61	25	6	77	12.0%	2.00 [0.16, 3.84]
McGrath 2016	24	6	29	23	6	34	8.3%	1.00 [−1.97, 3.97]
Picón-César 2021	23.48	6.15	70	22.19	6.82	20	7.4%	1.29 [−2.03, 4.61]
Rochon 2006	26	7	80	24	7	21	7.3%	2.00 [−1.36, 5.36]
Tertti 2013	27.1	2.3	87	25.7	3.2	23	13.6%	1.40 [0.01, 2.79]
Yogev 2011	26.5	2.8	93	25.9	5.3	31	11.6%	0.60 [−1.35, 2.55]
Total (95% CI)			1026			447	100.0%	2.64 [1.42, 3.86]

Heterogeneity: $Tau^2 = 2.34$; $Chi^2 = 26.42$, $df = 10$ ($P = 0.003$); $I^2 = 62\%$
Test for overall effect: $Z = 4.24$ ($P < 0.0001$)

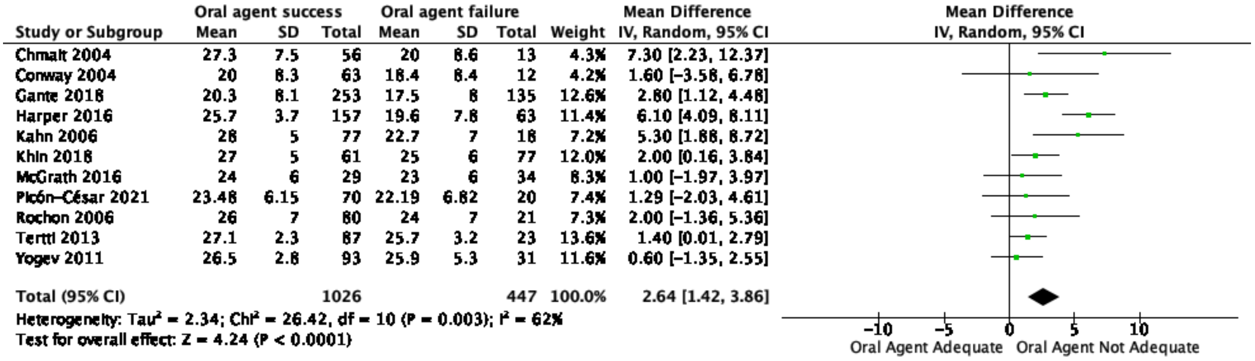

b)

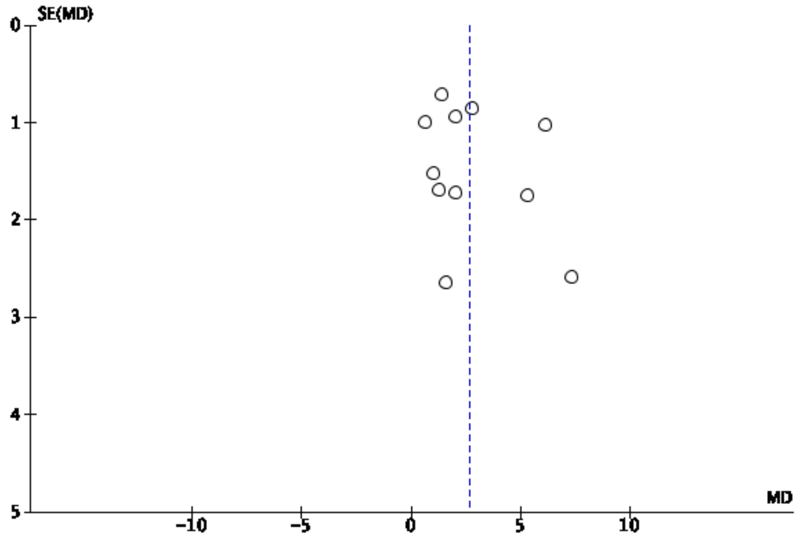

Supplementary Figure 25 Comparison of studies of whether oral pharmacological agent was adequate in controlling glucose or inadequate according to gestational age in weeks at initiation of oral pharmacological agent for treatment of gestational diabetes

Legend. Forest plot (a) and Funnel plot (b) for included studies comparing if lifestyle was adequate (no need for escalation to insulin) or not adequate (required escalation to insulin).

a)

	Oral agent success			Oral agent failure				Mean Difference
Study or Subgroup	Mean	SD	Total	Mean	SD	Total	Weight	IV, Random, 95% CI
Chmait 2004	29.7	6.6	56	23	6.9	13	9.4%	6.70 [2.57, 10.83]
Gante 2018	28.8	6.1	253	24.3	6.6	135	18.2%	4.50 [3.16, 5.84]
Kahn 2006	31	4	77	24	7	18	11.5%	7.00 [3.65, 10.35]
Khin 2018	29	4	61	26	6	77	17.1%	3.00 [1.33, 4.67]
McGrath 2016	28.9	4.9	29	25.3	5.8	34	13.8%	3.60 [0.96, 6.24]
Picón-César 2021	28.23	5.01	70	24.6	6.92	20	11.8%	3.63 [0.38, 6.88]
Yogev 2011	30.1	2.7	93	29.5	3.3	31	18.3%	0.60 [−0.68, 1.88]
Total (95% CI)			639			328	100.0%	3.79 [2.08, 5.51]

Heterogeneity: $Tau^2 = 3.76$; $Chi^2 = 27.06$, $df = 6$ ($P = 0.0001$); $I^2 = 78\%$
Test for overall effect: $Z = 4.33$ ($P < 0.0001$)

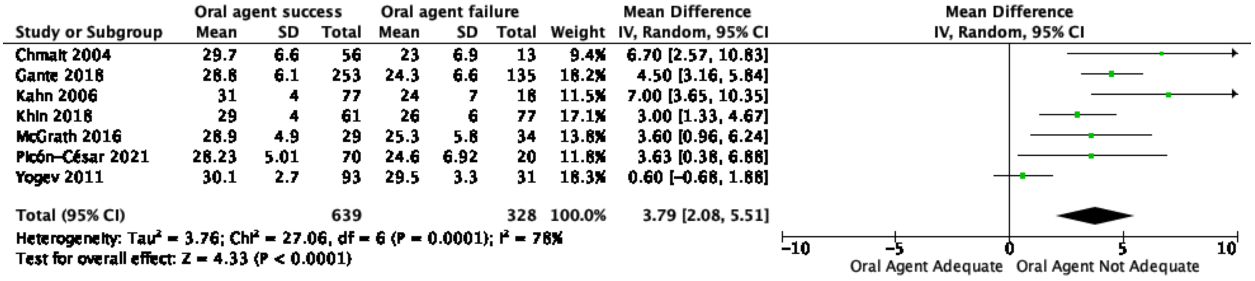

b)

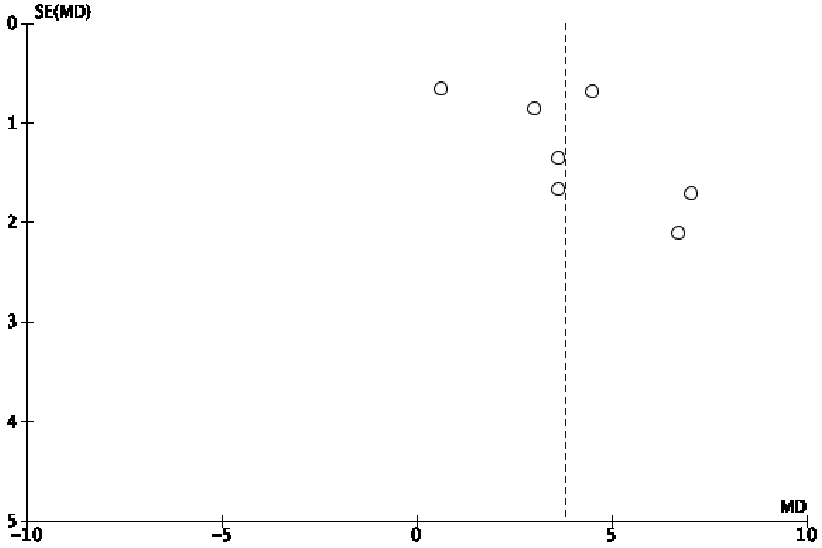

Supplement: Supplementary file 1 — Supplementary Information [file 43856_2023_371_MOESM1_ESM.pdf]
