# Supplementary material for: Precision gestational diabetes treatment: a systematic review and meta-analyses
Source: Commun Med (Lond). 2023 Oct 5;3:135. doi: 10.1038/s43856-023-00371-0 (PMC10550921; doi:10.1038/s43856-023-00371-0)
Supplement: Supplementary file 7 — Description of Additional Supplementary Files [file 43856_2023_371_MOESM7_ESM.pdf]

Description of Additional Supplementary Files

File Name: Supplementary Data 1
Description: Precision markers to enhance behavioural (diet and lifestyle) interventions
Legend: [b] Studies included in narrative synthesis

File Name: Supplementary Data 2
Description: Precision markers for escalation of pharmacological interventions to achieve target glucose levels
Legend: *Studies in each subgroup listed in alphabetical order of first author; [a] Studies included in meta-analyses; [b] Studies included in narrative synthesis; **Wong 2011 and 2012 contain overlapping data; ***Huhtala 2020 is secondary analyses of Tertti 2013

File Name: Supplementary Data 3
Description: Likelihood of oral agent failure by agent (glyburide or metformin).
Legend: OR (95% CI) Odds ratio (95% Confidence Interval)

File Name: Supplementary Data 4
Description: Narrative summary of studies not included in the meta-analysis
Legend: Table details key precision markers reported in the listed studies. Studies in each sub-section are in alphabetical order.
