# Supplementary material for: Precision gestational diabetes treatment: a systematic review and meta-analyses
Source: Commun Med (Lond). 2023 Oct 5;3:135. doi: 10.1038/s43856-023-00371-0 (PMC10550921; doi:10.1038/s43856-023-00371-0)
Supplement: Supplementary file 8 — Reporting Summary [file 43856_2023_371_MOESM8_ESM.pdf]

nature portfolio

Corresponding author(s): Rebecca Reynolds

Last updated by author(s): Jul 26, 2023

Reporting Summary

Nature Portfolio wishes to improve the reproducibility of the work that we publish. This form provides structure for consistency and transparency in reporting. For further information on Nature Portfolio policies, see our Editorial Policies and the Editorial Policy Checklist.

Statistics

For all statistical analyses, confirm that the following items are present in the figure legend, table legend, main text, or Methods section.

n/a	Confirmed	
☐	☒	The exact sample size (*n*) for each experimental group/condition, given as a discrete number and unit of measurement
☐	☒	A statement on whether measurements were taken from distinct samples or whether the same sample was measured repeatedly
☐	☒	The statistical test(s) used AND whether they are one- or two-sided<br>*Only common tests should be described solely by name; describe more complex techniques in the Methods section.*
☐	☒	A description of all covariates tested
☐	☒	A description of any assumptions or corrections, such as tests of normality and adjustment for multiple comparisons
☐	☒	A full description of the statistical parameters including central tendency (e.g. means) or other basic estimates (e.g. regression coefficient) AND variation (e.g. standard deviation) or associated estimates of uncertainty (e.g. confidence intervals)
☐	☒	For null hypothesis testing, the test statistic (e.g. F, t, r) with confidence intervals, effect sizes, degrees of freedom and P value noted<br>*Give P values as exact values whenever suitable.*
☐	☒	For Bayesian analysis, information on the choice of priors and Markov chain Monte Carlo settings
☐	☒	For hierarchical and complex designs, identification of the appropriate level for tests and full reporting of outcomes
☐	☒	Estimates of effect sizes (e.g. Cohen's *d*, Pearson's *r*), indicating how they were calculated

Our web collection on statistics for biologists contains articles on many of the points above.

Software and code

Policy information about availability of computer code

Data collection	Covidence software Veritas Health Innovation, Australia, available at www.covidence.org) was used for the screening of title, abstracts and selection of full texts for these reviews.
Data analysis	Statistical analyses were performed using Review Manager software [RevMan, Version 5.4.1, The Cochrane Collaboration, Copenhagen, Denmark]

For manuscripts utilizing custom algorithms or software that are central to the research but not yet described in published literature, software must be made available to editors and reviewers. We strongly encourage code deposition in a community repository (e.g. GitHub). See the Nature Portfolio guidelines for submitting code & software for further information.

Data

Policy information about availability of data

All manuscripts must include a data availability statement. This statement should provide the following information, where applicable:

- Accession codes, unique identifiers, or web links for publicly available datasets
- A description of any restrictions on data availability
- For clinical datasets or third party data, please ensure that the statement adheres to our policy

Data are available through contact with the corresponding author.

Research involving human participants, their data, or biological material

Policy information about studies with human participants or human data. See also policy information about sex, gender (identity/presentation), and sexual orientation and race, ethnicity and racism.

Reporting on sex and gender	This has been reported
Reporting on race, ethnicity, or other socially relevant groupings	We include a statement about pregnancy and the term woman/women:<br>As part of the diabetes scientific community, we are sensitive in using inclusive language, especially in relation to gender. However, the vast majority of original studies that the GDM precision medicine working groups reviewed used 'women' as their terminology to describe their population, as GDM per definition occurs in pregnancy which can only occur in individuals that are female at birth. To be consequent consistent with the original studies defined populations, we use the word 'women' in our summary of the evidence, current gaps and future perspectives, but fully acknowledge that not all individuals who experienced a pregnancy may self-identify as 'women' at all times over their life course.
Population characteristics	This is included
Recruitment	This was a secondary analyses and synthesis of published data so no new recruitment was done
Ethics oversight	Ethical approval was not required as these were secondary studies using published data.

Note that full information on the approval of the study protocol must also be provided in the manuscript.

Field-specific reporting

Please select the one below that is the best fit for your research. If you are not sure, read the appropriate sections before making your selection.

☒ Life sciences ☐ Behavioural & social sciences ☐ Ecological, evolutionary & environmental sciences

For a reference copy of the document with all sections, see nature.com/documents/nr-reporting-summary-flat.pdf

Life sciences study design

All studies must disclose on these points even when the disclosure is negative.

Sample size	This was a systematic review so the sample size was determined by our inclusion criteria.
Data exclusions	We excluded studies with a total sample size <50 participants to ensure sufficient data to interpret the effect of precision markers. We also excluded studies published before or during 2004, in order to consider studies with standard care similar to ACHOIS, thus that studies were clinically relevant to today's population.s
Replication	Replication is not possible with a systematic review.
Randomization	Titles, Abstracts and full texts for review were randomly assigned to reviewers through the covidence software.
Blinding	Blinding was not possible due to this being a systematic review.

Reporting for specific materials, systems and methods

We require information from authors about some types of materials, experimental systems and methods used in many studies. Here, indicate whether each material, system or method listed is relevant to your study. If you are not sure if a list item applies to your research, read the appropriate section before selecting a response.

Materials & experimental systems

n/a	Involved in the study	
☒	☐	Antibodies
☒	☐	Eukaryotic cell lines
☒	☐	Palaeontology and archaeology
☒	☐	Animals and other organisms
☐	☒	Clinical data
☒	☐	Dual use research of concern
☒	☐	Plants

Methods

n/a	Involved in the study	
☒	☐	ChIP-seq
☒	☐	Flow cytometry
☒	☐	MRI-based neuroimaging

Clinical data

Policy information about clinical studies

All manuscripts should comply with the ICMJE guidelines for publication of clinical research and a completed CONSORT checklist must be included with all submissions.

Clinical trial registration	This was a systematic review and so was registered on Prospero
Study protocol	PROSPERO registration IDs CRD42022299288 and CRD42022299402
Data collection	The data collection was done during 2022
Outcomes	Predefined outcomes were agreed by consensus and pre-registered within the Prospero protocols
